# Proteomic Approaches to Study Cysteine Oxidation: Applications in Neurodegenerative Diseases

**DOI:** 10.3389/fnmol.2021.678837

**Published:** 2021-06-09

**Authors:** Trong Khoa Pham, Weronika A. Buczek, Richard J. Mead, Pamela J. Shaw, Mark O. Collins

**Affiliations:** ^1^Sheffield Institute for Translational Neuroscience (SITraN), University of Sheffield, Sheffield, United Kingdom; ^2^Department of Biomedical Science, University of Sheffield, Sheffield, United Kingdom

**Keywords:** oxidation, oxidative stress, proteomics, neurodegenerative disease, amyotrophic lateral sclerosis (ALS), Alzheimer’s disease

## Abstract

Oxidative stress appears to be a key feature of many neurodegenerative diseases either as a cause or consequence of disease. A range of molecules are subject to oxidation, but in particular, proteins are an important target and measure of oxidative stress. Proteins are subject to a range of oxidative modifications at reactive cysteine residues, and depending on the level of oxidative stress, these modifications may be reversible or irreversible. A range of experimental approaches has been developed to characterize cysteine oxidation of proteins. In particular, mass spectrometry-based proteomic methods have emerged as a powerful means to identify and quantify cysteine oxidation sites on a proteome scale; however, their application to study neurodegenerative diseases is limited to date. Here we provide a guide to these approaches and highlight the under-exploited utility of these methods to measure oxidative stress in neurodegenerative diseases for biomarker discovery, target engagement and to understand disease mechanisms.

## Introduction

Post-translational modifications (PTMs) of cysteine (Cys) residues play a crucial role in protein function and cellular homeostasis. In particular, cysteine residues are sensitive to oxidative stress and are subject to a range of reversible and irreversible oxidations. Many mass spectrometry (MS)-based proteomics approaches have been developed to identify and measure levels of oxidized cysteine residues (Oxi-Cys) in a range of biological systems. These quantitative proteomics approaches include the analysis of reversible, irreversible and specific sub-types of Oxi-Cys PTMs. Oxidative stress is a pathological feature of a range of neurodegenerative diseases, but proteomics methods to study Oxi-Cys PTMs in this context are underexploited. In this review, we survey state-of-the-art approaches for Oxi-Cys PTMs analysis to guide researchers who wish to exploit proteomics to probe cysteine oxidation in neurodegenerative disease.

### Cysteine Oxidation

Among four common sulfur-containing amino acids, homocysteine, taurine, methionine and cysteine, only the latter two are incorporated into proteins. Although both of these two play essential roles in cell metabolism, cysteine plays a crucial role in the structure of proteins and protein-folding pathways because of its ability to form intra- (inside a protein) and inter-chain (different proteins) linkages with other cysteine residues (Brosnan and Brosnan, 2006). Only 2.3% of the human proteome encodes Cys residues, but their activities vary depending on their states, mainly reduced or oxidized forms, within proteins. Many attempts have been performed to characterize oxidation states of specific Cys residues, but success has been limited because of the low abundance of oxidatively modified Cys in biological systems and the diversity of its oxidative products. The oxidation of other amino acids also plays an important role in neurodegenerative diseases (such as methionine and tyrosine oxidation) (Shi and Carroll, 2020), but they are not the focus of this review.

**FIGURE 1:**
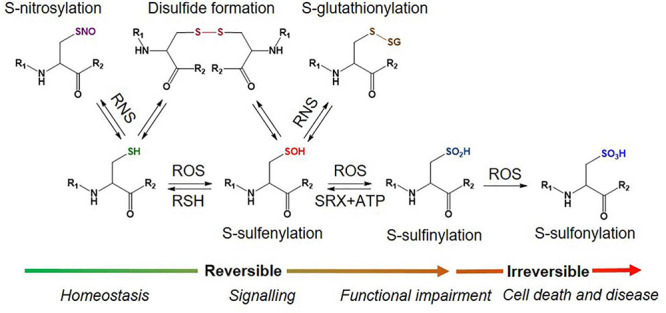
Different types of Oxy-Cys PTMs occurring in proteins.

There are two primary levels of Oxi-Cys residues in proteins depending on the level of cellular oxidative stress, reversible and irreversible Cys oxidations. The reversible oxidation of Cys, such as *S*-nitrosylation (*S*-NO), *S*-sulfenylation (*S*-OH), *S*-glutathionylation (*S*-SG), and disulphide formation (*S*–*S*) (Figure 1), is found under low oxidative conditions and acts to modulate protein function (Dalle-Donne et al., 2005). However, at higher levels of oxidative stress, the oxidative state of Cys is elevated and harder to reverse (*S*-sulfinylation, *S*-O_2_H) or becomes irreversible (*S*-sulfonylation, *S*-O_3_H, or *S*-sulfinylation) (Figure 1), causing the loss of protein function (Murray and Eyk, 2012). The determination of dynamic changes in site-specific Cys oxidation will provide vital information to understand the distribution of redox balance states in neurodegenerative diseases and to characterize dysregulated oxidation in patients compared to healthy control (HC) groups. The reversible oxidation states of Cys may also reflect the fluctuations of cellular oxidation status, and both reversible and irreversible oxidation status of Cys may serve as biomarkers of neurodegenerative diseases associated with oxidative stress.

### Oxidative Stress in Neurodegeneration

Multiple lines of evidence point to a role for elevated oxidative stress in driving neuronal degeneration in neurodegenerative diseases such as Alzheimer’s disease (AD) (Markesbery and Carney, 1999), Parkinson’s disease (PD) (Puspita et al., 2017) and amyotrophic lateral sclerosis (ALS) (Barber and Shaw, 2010). We are still unsure whether oxidative stress is a cause or consequence of neuronal degeneration. In addition, many therapeutic approaches have been tested in clinical studies in an attempt to limit pro-oxidative mediated neuronal injury. However, few of these approaches, except Edaravone for ALS (Group, 2017), have been successful.

Oxidative stress has broadly been considered an imbalance in pro and anti-oxidant processes, leading to elevated pro-oxidant activity in cells, leading to cellular injury and death (Guo et al., 2013; Song et al., 2021). For example, a common denominator in neurodegenerative diseases is the presence of oxidatively damaged macromolecules in neurons and glia and other biosamples. However, oxidative stress is probably better understood as a disruption of the interplay between different inter-related cellular redox systems and their control (Jones, 2006). Therefore, to understand and therapeutically modulate redox control and signaling in neurodegeneration, it is critical to measure the status of these different redox systems. Cysteine redox cycling is a key component of these systems, and the study of this pathway has been relatively neglected to date. However, it is eminently accessible *via* proteomic approaches, enabling a global view of cysteine redox regulation.

The refinement of measures of complex redox biology in the central nervous system (CNS) directly in patients, as disease progresses, would greatly facilitate both the understanding of the role that redox regulation plays in neurodegeneration, but importantly would also enable identification of novel biomarkers for evaluating therapeutic interventions targeted at oxidative stress. So-called target engagement biomarkers are considered critical in the development of novel therapeutics (Durham and Blanco, 2015).

The misfolding and aggregation of amyloid-beta (Aβ) and tau in AD and superoxide dismutase type-1 (SOD1) in ALS results in a progressive loss of specific populations of neurons that is strongly associated with mitochondrial dysfunction, neuroinflammation and excitotoxicity (Andersen, 2004; Guo et al., 2013; Song et al., 2021). These pathological processes lead to increased generation of free radicals, redox imbalance which further impairs cellular function, leading to neuronal cell death.

Protein aggregates are formed by oxidatively altered proteins that misfold and accumulate. Multiple PTMs may contribute to protein misfolding; one of these is Cys oxidation (Olzscha, 2019). Several mutations in the Cu/Zn superoxide dismutase gene (SOD1) cause familial amyotrophic lateral sclerosis (FALS). There is also evidence to suggest that oxidized wild-type SOD1 adopts a conformation similar to mutant SOD1, which could be pathogenic in sporadic ALS (Bosco et al., 2010). In another study, the oxidation of C_111_ on SOD1 *via* glutathionylation (*S*-SG) was found to decrease the stabilization of SOD1 dimer, increasing the potential for the unfolding of the monomer and subsequent aggregation and, as a result, leads to loss of SOD1 activity and promotion of cell death (Redler et al., 2011). Peroxiredoxins (Prxs), enzymes that neutralize reactive oxygen/nitrogen species, have been implicated in several neurodegenerative diseases, including AD and PD. They act to reduce peroxynitrite and other hydroperoxides through a redox-sensitive Cys within their active sites; they reduce peroxide substrates *via* the formation of intramolecular disulphide bonds or oxidation to sulfinic acid or sulfonic acid (Hall et al., 2009). The protective function of PRDX2, the most abundant peroxiredoxin in neurons, against oxidative stress is inhibited by oxidation of the enzyme itself. *S*-NO of PRDX2 at the active-site residues Cys 51 and 172 inhibits the enzyme activity, the level of which is increased in human PD brains (Fang et al., 2007).

In AD, *S*-NO can have both neuroprotective and neurotoxic functions (Zhao et al., 2015). For example, *S*-NO of the *N*-methyl-D-aspartate (NMDA) receptor inhibits its overactivation and promotes neuronal survival (Nakamura and Lipton, 2007). In contrast, many proteins that are key to normal synaptic function are aberrantly *S*-nitrosylated in AD, which results in synaptic damage and neurodegeneration (Nakamura et al., 2013). Therefore, targeting protein *S*-NO can prove useful as a new therapeutic intervention for AD neurodegeneration.

Neurons are particularly susceptible to oxidative stress because of the high energy demand, which depends heavily on robust mitochondrial function. Under physiological conditions, mitochondria serve as a primary source of reactive oxygen species (ROS) in the cell and in normal conditions, the physiological antioxidant defense systems can neutralize these. However, mitochondrial dysfunction leads to increased ROS levels and has been implicated in AD pathology (Lin and Beal, 2006). Swerdlow and Khan (2004) and Swerdlow et al. (2014) proposed a ‘mitochondrial cascade hypothesis,’ which suggests that oxidative stress and amplified generation of ROS trigger Aβ aggregation and neurodegeneration. *In vitro* models of both AD and ALS provided evidence that mitochondrial fragmentation and dysfunction trigger ROS production by microglia, resulting in neuronal damage (Joshi et al., 2019). In a mouse model of AD, partial deficiency of manganese superoxide dismutase (MnSOD) is known to induce oxidative stress, elevated Aβ levels and plaque deposition (Li et al., 2004). In another AD mouse model, pharmacological inhibition of energy production was also amyloidogenic, and metabolic dysregulation has been suggested to be an early pathological event in AD (Velliquette et al., 2005). These results support the hypothesis that altered energy metabolism is a key mechanism in AD.

Studies on neuroinflammation have generated more evidence showing the role of ROS in AD. For example, activation of the NOD-like receptor family, pyrin domain containing 3 (NLRP3) inflammasome, has been widely implicated in various neurological disorders (Shao et al., 2018). One of the main features of AD is microglial activation, which enhances the release of pro-inflammatory molecules, including ROS, and contributes to the propagation of the disease. Aβ promotes the accumulation of mitochondrial ROS, which causes NLRP3 inflammasome activation (Wang et al., 2017). The activation of the NLRP3 inflammasome is thought to seed and spread the Aβ pathology within and between brain structures (Venegas et al., 2017). Moreover, there is strong evidence suggesting that NLRP3 inflammasome activation by ROS also drives tau pathology (Ising et al., 2019). Therefore, this example shows a clear connection between neuroinflammation and ROS production occurring in Aβ- and tau-related neurodegeneration. Since oxidative damage is associated with both healthy aging and disease, understanding the mechanisms of cellular oxidation status in aging and neurodegeneration may lead to new strategies for their prevention.

### Overview of Oxi-Cys Proteomics

In the field of Oxi-Cys proteomics, one of the main challenges lies in the sample preparation methods used to access (and preserve) the initial oxidation state of proteins within oxidative environments. A general protocol for MS-based Oxi-Cys proteomics includes two main steps: chemical labeling reduced and oxidized cysteine residues and MS-based analysis to identify and quantify peptides (bottom-up) or intact proteins (top-down) containing labeled cysteine residues. The key steps for sample preparation and labeling of cysteine-containing proteins consist of extracting proteins using appropriate buffers, blocking free reduced cysteine residues, reducing reversibly oxidized cysteines using either reducing reagents or targeting the type of oxidation of interest using suitable probes. Subsequently, labeled and/or purified proteins/peptides are analyzed by MS. For the bottom-up proteomics approach, labeled proteins are enzymatically digested into peptides (typically using trypsin) to generate a complex mixture of peptides, which is then analyzed directly by MS analysis or fractionated using HPLC to reduce the complexity of peptide mixture before MS analysis of each fraction. For top-down analysis, an intact protein is enriched/purified using suitable techniques before being directly analyzed and fragmented in a tandem mass spectrometer (Virág et al., 2020). As the intact protein is analyzed, the context of multiple PTMs and protein isoforms is retained (Tran et al., 2011), unlike bottom-up proteomics, where the connection between PTMs on different peptides is lost. However, sequence coverage is generally much lower in top-down versus bottom-up proteomics. Top-down proteomics is not currently well suited for the analysis of complex mixtures of proteins (Lorenzatto et al., 2015), and data analysis can be complex and time-consuming. As a result, this approach is not currently routinely used for high-throughput proteomic analysis, and only a few studies have used this approach to study protein oxidation (Tran et al., 2011; Ansong et al., 2013; Auclair et al., 2014; Reinwarth et al., 2014). Therefore, in this review, we will highlight the most currently used bottom-up proteomics approaches for the studies of Oxi-Cys proteomes.

## Chemical-Labeling Approaches for Oxi-Cys PTMs

There are many considerations for the experimental design for proteomic analysis of Oxi-Cys PTMs. These include the types of Cys-oxidations of interest, number of biological samples, selection of approaches (reactive thiols or oxidized Cys), incorporation of chemical tags, enrichment method, availability of antibodies, and type of quantitation (Gu and Robinson, 2016b). In Oxi-Cys based analysis, abundance changes in peptides containing oxidized Cys will directly reflect the oxidation level in a given sample/experiment. In thiol reactivity profiling experiments, abundance changes in reduced Cys will reflect the oxidative level of Cys inversely. Generally, a typical workflow for Oxi-Cys proteomics analysis in neurodegenerative diseases should cover:

• Types of Oxi-Cys PTMs of interest (global or selected PTMs as seen in Figure 1) to ensure that, in the case of selected Oxi-Cys, the (affinity) purification method is available and suitable.
• Quantitative approaches to be used, as this will determine the types of thiol alkylations, appropriate reagents, protein/peptide enrichment and labeling, MS [MS/MS^(n)^] operation, and cost-effectiveness.
• Type of MS instrument available for sample analysis.

**FIGURE 2:**
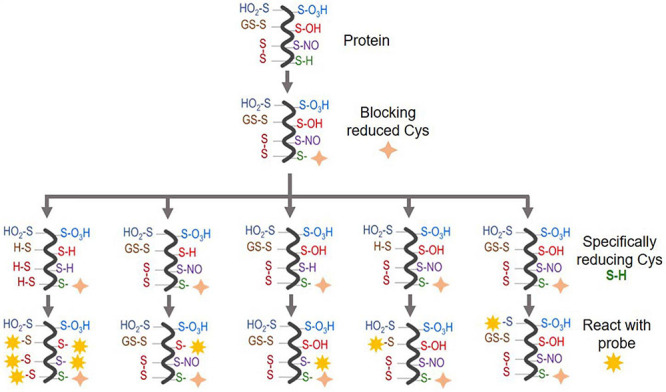
An overview of Oxi-Cys quantitative proteomics approaches for different types of Cys oxidations. Chemicals used for selective Oxi-Cys proteomics analysis. Free reduced Cys residues are firstly labeled with alkylating reagents (IAM, NEM, MMTS, *etc.*) while different PTMs of reversible Oxi-Cys residues are selectively alkylated by specific reducing regents; for example, DTT/TCEP for reducing all reversible Cys, arsenite for *S*-sulfenylation (*S*-OH), ascorbate for *S*-nitrosylation (*S*-NO), glutaredoxin for *S*-glutathionylation (*S*-SG). These newly formed reduced Cys residues then, theoretically, can be labeled by different alkylating reagents or specific probes coupled with tags for purification/enrichment. The quantitation is performed based on comparing intensities of tags from diseased compared to control groups. *S*-OH probes include: dimedone and bicyclononyne BCN; *S*-O_2_H probes include: *C*-nitroso esters, diazenes and *S*-nitrosothiols as detailed in Figures 5A,B.

These key factors will affect the selection of appropriate approaches, MS (MS/MS) mode operation, and downstream data processing. We have summarized the main proteomic approaches for different types of Oxi-Cys PTM analyses in Figure 2, and their applications, advantages and disadvantages are shown in Table 1 and Supplementary Table 1, respectively. The details of these approaches are discussed in the following sections.

**TABLE 1:** Advantages and disadvantages of current Oxi-Cys proteomic approaches.

**Approach**	**Advantages**	**Disadvantages**	**References**
SICyLIA	Simple protocol. Saves time and cost. Increased detection of low abundance oxidized Cys. High oxi-proteome coverage. Accurate quantitation.	Cannot distinguish different forms of reversible oxidized Cys. Need to control IAM reaction carefully to reduce the loss of deuterium in heavy IAM. Only two samples can be compared in a single experiment.	van der Reest et al., 2018
IodoTMT	Multiple samples (up to 6 samples per experiment).	Low selectivity. Antibody based purification.	Qu et al., 2014
cysTMTRAQ	Multiplexing analysis. Analyze both Oxi-Cys dynamics and protein-level changes in a single experiment. Can perform either Cys enrichment or not. Determine stoichiometry of redox modifications. High confidence and accuracy for oxi-Cys proteome analysis.	Costly and complicated protocol. Interference of both TMT and iTRAQ ion reporters cause ratio compression.	Parker et al., 2015
isoTOP-ABPP	Measure/monitor directly functions of enzymes in native biological systems. Exact sites of Cys reactivity. Low (μM) of IA used to allow differentiate the extent of alkylation. The isoTOP-ABPP ratio is independent with both peptide and protein abundances. Proteome-wide profiles of Cys reactivity in complex biological systems.	Incomplete precipitation of proteins after click chemistry (∼50% of total proteins). Low accurate characterization of too large or too short peptides. Combination of digested enzymes need to be used to increase proteome coverage. Limit in software that can be used for data analysis. Long protocol.	Weerapana et al., 2007; van der Reest et al., 2018
QTRP	High yield of biotinylated as click chemistry reaction performed on a peptide level.	Control concentration of IPM for alkylation.	van der Reest et al., 2018
DiaAlk	Measure directly *S*-sulfinylation and *S*-sulfenylation. High selectivity of *S*-sulfinated peptides/proteins.	Applicable for 2 biological samples only. Complicated sample preparation. Prolonged and costly protocol. Availability of chemicals. Many chemical reactions Not optimized to measure global level of *S*-sulinylated Cys directly in cells. Requires biotin based purification and photo-cleavage to release tags.	Akter et al., 2018
OxiMRM	Benefit for low abundance oxidized proteins. Detect/measure both reversibly and irreversibly oxidized Cys.	Two different alkylating steps. Many precipitation steps. Need specific antibodies. Low number of peptides detected.	Held et al., 2010
UPLC-pSRM	Label free approach. No affinity purification required. Detect/measure irreversibly oxidized Cys.	Underestimation of low abundance oxidized protein detection. Requires specific antibody for immunoaffinity purification. Prior knowledge of targets required.	Sherrod et al., 2012
Cys-DIA	High coverage of Cys proteome.	Only used with certain MS instruments. Need to run DDA to create customized database. Samples run individually does not support multiplexing. Need to purify Cys.	Tahir et al., 2020

There are two main types of Oxi-Cys analysis: global Oxi-Cys and selective Oxi-Cys measurement (Figure 2). Traditionally, for any relative quantitative analysis of Oxi-Cys, at least two different biological groups are required. The global quantitative Oxi-Cys analysis can be performed based on the ratio of Cys-containing peptide XIC (extracted ion chromatogram) intensities detected by MS (either reduced or oxidized Cys intensities) between samples. This type of analysis includes two different alkylating steps (e.g., IAM and NEM), stable isotopic labeling (SICyLIA), resin-assisted capture (RAC) combined with isobaric labeling reagents (TMT, iTRAQ, and iCAT). Irreversible cysteine oxidations are typically stable during sample processing and LC-MS conditions; thus, direct analysis of these modifications in a complex sample is feasible.

### Differential Alkylation

#### Chemically Distinct Alkylation Reagents

**FIGURE 3:**
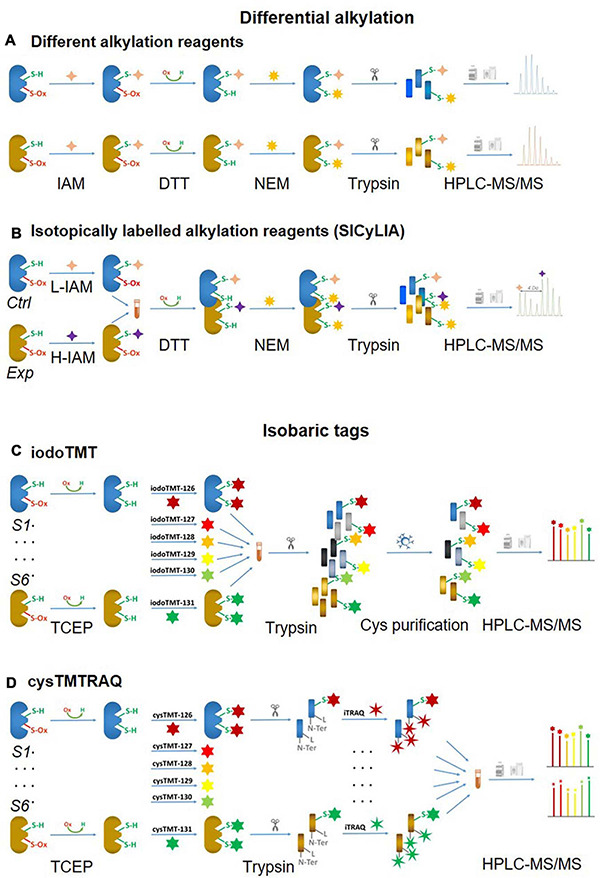
Non-click chemistry-based thiol-reactive workflows. Different alkylation methods: **(A)** two chemically distinct alkylation reagents and **(B)** SICyLIA; isobariac tags: **(C)** iodoTMT and **(D)** cysTMTTRAQ.

One of the biggest challenges of reversible Oxi-Cys analysis is to preserve the endogenous redox state. This can be affected by either oxidation or reduction of Cys during sample preparation leading to inaccurate cellular redox balance measurement. To minimize the artefactual oxidation, alkylating reagents such as Iodoacetamide (IAM) or *N*-ethylmaleimide (NEM) are added at the first step during protein denaturation to alkylate reduced Cys residues (McDonagh et al., 2014). This is a crucial step for relative quantification, and it is required that the alkylating reagent should completely block all reduced Cys. Subsequently, all reversible Oxi-Cys residues are reduced by reducing agents, such as dithiothreitol (DTT), dithiobutylamine (DTBA), β-mercaptoethanol (BME), or tris(2-carboxyethyl)phosphine (TCEP) to generate free sulfhydryl groups. This is followed by the second alkylation step, where newly formed sulfhydryl groups are chemically blocked with an alkylating reagent distinct from that used in the first step [e.g., NEM, IAM, 2-chloroacetamide, iodoacetic acid (IAA), 4-vinylpyridine, methanethiosulfonate (MMTS)] (McDonagh et al., 2014). Proteins are then enzymatically digested, and the generated peptides can then be fractionated, if necessary, *via* an off-line HPLC and fractions analyzed by LC-MS/MS (Figure 3A). Intensities of reduced Cys and Oxi-Cys-containing peptides from samples are compared to estimate the ratio of Oxi-Cys. This approach is relatively straightforward, and from the same analysis, protein expression measurement can be performed using label-free quantification of non-cysteine containing peptides. However, given that this approach does not include a cysteine enrichment step, the coverage of Cys-containing peptides for complex samples may be lower than that achievable using other methods that do incorporate an enrichment step.

#### Stable Isotopic Labeling With Iodoacetamide (SICyLIA)

**FIGURE 4:**
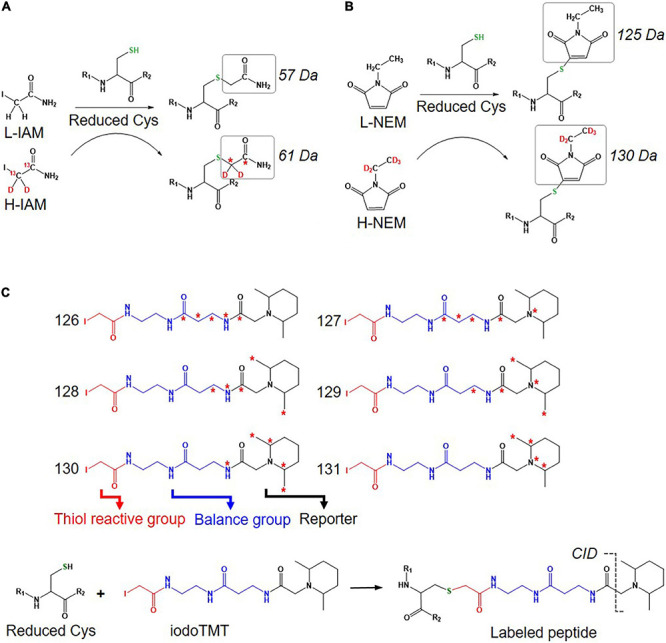
Isotope labeled reagents and probes used for Oxi-Cys proteomics. Isotopic chemicals and their adding extra mass for PTM search: **(A)** L-IAM and H-IAM, **(B)** L-NEM, and H-NEM, **(C)** iodoTMT reagents and the cleavage site to release ion reporters by CID during MS/MS analysis.

An approach using stable isotope cysteine labeling with IAM, termed SICyLIA, has been recently developed to investigate global Oxi-Cys proteomes (van der Reest et al., 2018). This approach utilizes light or heavy isotope-labeled IAM to differently label reduced Cys thiols between two groups (e.g., control and experimental). A detailed workflow of this approach is shown in Figure 3B. During denaturing, free reduced Cys residues in control and experimental samples are alkylated using either light (^12^C_2_H_4_INO) or heavy (^13^C_2_D_2_H_2_INO) IAM, respectively which results in the addition of a carbamidomethyl group (CAM), adding an extra of 57 or 61 Da to cysteine residues (Figure 4A); or free reduced Cys can be also alkylated with either light or heavy NEM (Figure 4B). These two samples are then combined and reduced using DTT; newly reduced Cys generated from reversible Oxi-Cys are alkylated with NEM and subjected to tryptic digestion to generate labeled peptides. The mixture of these peptides is then fractionated off-line by a high pH reversed-phase chromatography to reduce the complexity of the peptide mixture to increase proteome coverage. Peptide fractions of labeled peptides are then analyzed by LC-MS/MS. The ratio (of signal intensity) between heavy and light IAM-labeled Cys-containing peptides will estimate reduced cysteine levels between two samples with an assumption that any decrease in CAM-modified peptides will reflect an increase in the level of oxidation (van der Reest et al., 2018). Given that the reduced Cys portion of proteins is more abundant in cells, this approach does not require an enrichment step and allows enhanced detection of oxidation changes in cells (van der Reest et al., 2018). However, this approach cannot distinguish specific forms of reversible Oxi-Cys, as all reversible Oxi-Cys residues are labeled and analyzed.

### Isobaric Tags

#### Iodoacetyl Isobaric Tandem Mass Tags – IodoTMT

Iodoacetyl isobaric tandem mass tags (iodoTMT) allows the pooling and analysis of up to six samples in a single Oxi-proteomics experiment. The iodoTMTsixplex reagents consist of six different isobaric isomers that can react with the sulfhydryl (-SH) group of protein *via* irreversible labeling step by forming an iodoacetyl-activated covalent bond (Bomgarden et al., 2012). The workflow of this approach is presented in Figure 3C, and the chemical structures of iodoTMTsixplex reagents with the distribution of isotopes and their reaction with reduced Cys are shown in Figure 4C. Each iodoTMT reagent contains three distinctive groups (Figure 4C), including a reporter (used for quantitation), mass normaliser (balance group) and a Cys reactive group (to react with a sulfhydryl group) (Bomgarden et al., 2012). Different iodoTMT reagents can be used to label either reduced or oxidized Cys residues (reduced before labeling) depending on the design of the experiment. The ion reporters (isobaric mass tags) are cleaved during MS2 (fragmentation) analysis generating unique reporter ions ranging from 126 – 131 *m/z* (Figure 4C). Their intensities in fragmentation spectra are then used to calculate the level of Cys oxidation from the samples (Bomgarden et al., 2012).

This approach can be used for either global Cys (reduced and reversible oxidized forms) or Oxi-Cys analyses. If it is used for global Cys analysis, all proteins are denatured and reduced with TCEP to ensure that all reversible Oxi-Cys are reduced. All initially and newly reduced Cys-containing proteins in each sample are then labeled with appropriate iodoTMT reagent; up to six samples can be labeled with six specific iodoTMT reagents (Figure 3C). If an iodoTMT approach is used for analyzing reversible Oxi-Cys, proteins are firstly denatured in the presence of IAM to block reduced Cys; the excess IAM is removed via trichloroacetic acid (TCA) precipitation of proteins. Subsequently, proteins are reduced with DTT to covert reversible Oxi-Cys to free thiols that are then labeled with iodoTMT reagents. All labeled proteins are pooled and precipitated by TCA to remove the excess iodoTMT reagents, followed by tryptic digestion (Bomgarden et al., 2012). Labeled iodoTMT peptides are enriched using immobilized anti-TMT antibody resin and eluates analyzed by LC-MS/MS. The intensity of ion reporters generated during MS2 analysis will be used for relative quantitation of Oxi-Cys while other MS2 information is used for peptide/protein sequencing.

This iodoTMT approach can also be used for selective Oxi-Cys analysis. Qu et al. (2014) combined biotin switch technique (BST) [adopted from (Jaffrey et al., 2001), see section Biotin Switch Technique (BST) for details] with iodoTMT labeling to improve the iodoTMT approach, such as robust workflow for *S*-NO PTM analysis, effective peptide digestion, unambiguous *S*-NO-Cys mapping, and enhanced high throughput (Qu et al., 2014). In brief, Cys residues containing free thiols are alkylated with MMTS, while nitrosylated Cys residues are selectively reduced with ascorbate and labeled with iodoTMT reagents. Six different iodoTMT labeled samples are then combined, reduced (by DTT) and alkylated (by IAM), digested by trypsin. Labeled peptides are enriched by anti-TMT antibody resin before being analyzed by LC-MS/MS. The enrichment efficiency of iodoTMT labeled peptides is low but optimization of elution conditions has increased the efficiency from 6% using standard conditions to 21% (Qu et al., 2014). In theory, iodoTMT can be used for the investigation of most common reversible Oxi-Cys by a combination of selected methods for enrichment of different kinds of Oxi-Cys (as shown in Figure 2) and iodoTMT for labeling of interested Oxi-Cys PTM.

#### Cysteine-Reactive Tandem Mass Tag (cysTMT) and Isobaric Tags for Relative and Absolute Quantitation (iTRAQ) – cysTMTRAQ

Conventional multiplexing approaches such as iodoTMT, which provide a readout of the changes in thiol redox state, do not allow measurement of global changes in protein expression. To overcome this, the double-labeling approach termed cysTMTRAQ combining two different isobaric tag approaches, the cysTMT and iTRAQ, was developed (Parker et al., 2015). The cysTMT approach utilizes two different isobaric tag groups with two different purposes: the cysTMT tags are used to label thiol groups on Cys in up to six samples (126, 127, 128, 129, 130, and 131 *m/z*), while the iTRAQ tags are used to label N-terminal and lysine residues of peptides in up to six various samples (114, 115, 116, 117, 119, and 121 *m/z*). This approach, therefore, allows the simultaneous measurement of the changes in Cys oxidation (from cysTMT) and changes in global protein expression (from iTRAQ). This allows cysteine oxidation data to be normalized to protein expression data to correct for changes in protein turnover (Parker et al., 2015). To enhance the coverage of Oxy-Cys proteomes, the enrichment of labeled proteins can be performed using a specific anti-TMT antibody after two labeling steps, coupled with HPLC based fractionation before MS analysis. The workflow of this approach is shown in Figure 3D, and its benefits are shown in Table 2. Although this approach offers many advantages, one of the main problems is the ratio compression in quantitation caused by the interference of both TMT and iTRAQ ion reporters; however, the use of advanced modes of data acquisition such as synchronous precursor selection (SPS) implemented in the current generation of Orbitrap analyzers can solve this issue (Erickson et al., 2015).

**TABLE 2:** List of (potential and confirmed) biomarkers in neurodegenerative diseases from Oxy-Cys proteomics studies.

**Neurodegenerative diseases**	**Human specimens. Sample sizes**	**Method used**	**PTMs**	**Biomarkers**	**Cys sites**	**References**
Multiple sclerosis	CSF	Modified proteomics approach (No reduction and alkylation applied) and a targeted database search		Extracellular Superoxide dismutase (ECSOD) α1-antitrypsin (A1AT) Phospholipid transfer protein (PLTP) Alpha-2-HS-glycoprotein Ectonucleotide pyrophosphate (ENPP-2) Gelsolin Interleukin-18 (IL-18) Ig heavy chain V III region POM	195 232 318 340 773 304 38 22	Srivastava et al., 2019
	7 MS patients (2 males + 5 females), ages 35.6 ± 10.5					
	5 idiopathic intracranial hypertension (IIH) patients used as control group (1 male + 4 females), age 26.2 ± 7.6					
AD	CSF	Online immunoaffinity chromatography -mass spectrometry of intact protein		Transthyretin (TTR) isoforms (TTR-Cys10-Cys (cysteinylated), TTR-Cys10-CysGly (cysteine-glycinylated), and TTR-Cys10-SG (glutathionylated)/)	10	Poulsen et al., 2014
	AD 37 patients					
	Mild cognitive impairment (MCI) 17					
	Normal pressure hydrocephalus (NPH)					
	Healthy control (HC) 7					
AD and PD	Human brain tissues	2D gel, immunostaining and MS	*S*-sulfonylation	Targets: sulfonic acid UCH-L1	220	Choi et al., 2004
AD	Inferior parietal lobule from patients	2D-Oxyblots and MS	*S*-glutathionylation	Deoxyhemoglobin, α-crystallin B, α-enolase Glyceraldehyde phosphate dehydrogenase (GAPDH)		Newman et al., 2007
AD	Hippocampus, substantia nigra and cortex, AD patients (*n* = 9) control (*n* = 5)	2D-Oxyblot and MS	*S*-NO	Voltage-dependent anion-selective channel protein 2 (VDAC2). Superoxide dismutase [Mn] (SOD2). Fructose-bisphosphate aldolase C (ALDOC).		Zahid et al., 2014
AD	Human Hippocampus	2D-Oxyblots and MS	*S*-OH, *S*-O2H, *S*-O3H Sulfenic acid, sulfinic acid, and sulfonic acid	Pin1	113	Chen et al., 2015
Human Prion Diseases	Human cortex and cerebellum	BST, avidin enrichment, iTRAQ, gel-free	*S*-NO	1509 *S*-nitrosylated proteins (*SNO*-proteins) were identified		Chen et al., 2016
AD	Human cortical brain Tissues *n* = 30 healthy control, *n* = 30 MCI and *n* = 30 AD	Immunoblots, IodoTMT	*S*-NO	Myelin-oligodendrocyte glycoprotein (MOG) Glutathione *s*-transferase Mu 3 (GSTM3) Four and a half LIM domains protein 1 (FHL1)	53, 127 3 65	Wijasa et al., 2020

### Click Chemistry Approaches

**FIGURE 5:**
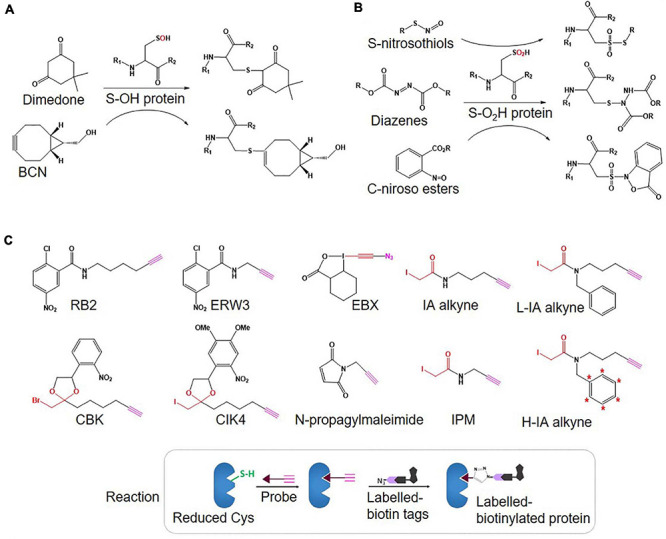
Structure of probes used for selective Oxi-Cys (**A:** *S*-OH and **B:** *S*-O_2_H) and click chemistry-based **(C)** approaches.

**FIGURE 6:**
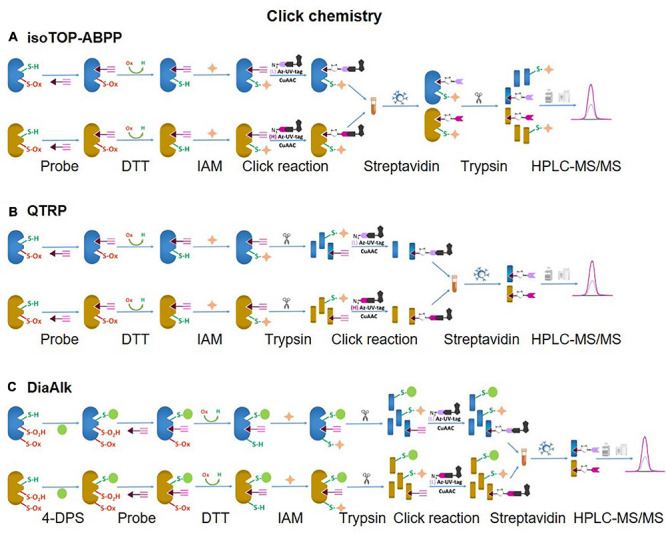
Click chemistry thiol-reactive workflows. **(A)** isoTOP-ABPP; **(B)** QTRP; **(C)** DiAlk. The Oxy-Cys quantitation of these approaches is based on MS1 level.

Unlike SiCyLIA or iodoTMT, in which different isotopic labeling reagents directly react with Cys residues in proteins without any other further reactions, click chemistry-based approaches (DiaAlk, TOP-ABPP) employ two reactions. Firstly, a probe (containing specified alkyne and an IAM group) will react with the thiol group in a protein (*via* IAM group) to form the probe-protein intermediate. This is then biotinylated with isotope labeled–biotin tags using a click chemistry reaction (*via* alkyne group) to allow subsequent purification of tagged proteins using avidin/streptavidin resins. Common probes used for selective Oxy-Cys proteomics analysis and click chemistry approaches are shown in Figure 5. Proteins labeled with either light or heavy isotope tags are usually tryptically digested to generate labeled peptides. Subsequently, the biotin group in these labeled peptides will be cleaved off by either enzymes (isoTOP-BPP approach) or UV (QTRP and DiaAlk approaches) at a specific wavelength, allowing labeled peptides to be readily analyzed by LC-MS/MS (see Figure 6). Based on signals of the (light and heavy) tag-labeled peptides obtained at MS1 level, a ratio of light/heavy (or versa) can be calculated to estimate the oxidation level of Cys in different samples/conditions.

#### Isotopic Tandem Orthogonal Proteolysis-Activity-Based Protein Profiling (isoTOP-ABPP)

**FIGURE 7:**
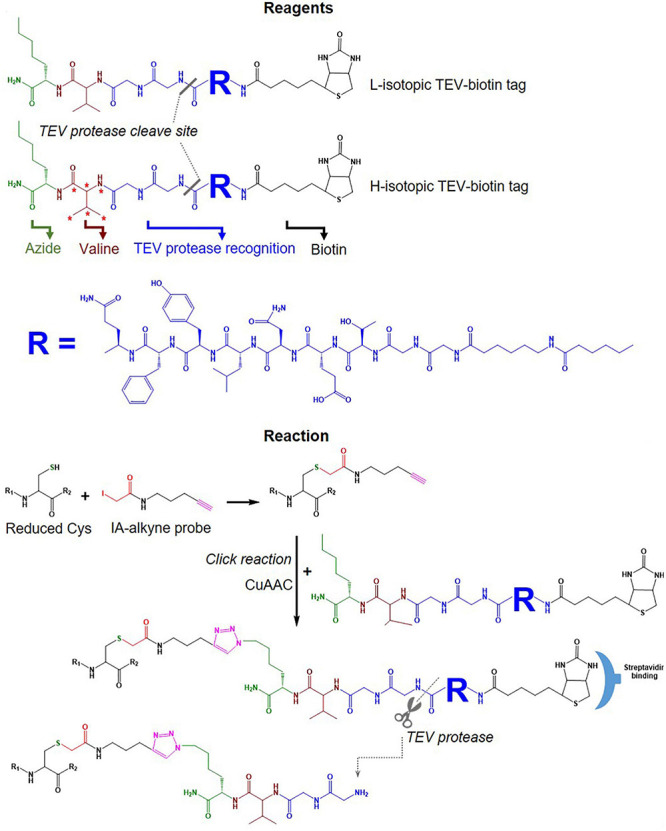
Tags used in the isoTOP-ABPP workflow. Structure of light and heavy isotopic TEV-biotin tags used in isoTOP-ABP, their reaction with reduced Cys, purification using streptavidin and release of labeled proteins using TEV protease.

IsoTOP-ABPP was developed as an advanced approach to quantify/measure the reactivity of Cys residues directly in native biological samples. It combines two different strategies; tandem orthogonal proteolysis (TOP) and activity-based protein profiling and has been used to predict functions of cysteine residues in proteomes (Weerapana et al., 2007). This approach consists of three main steps, alkylating reduced Cys in proteins with a probe, click chemistry-based incorporation of isotopically-labeled cleavable tags, protein purification and on-bead digestion (Weerapana et al., 2007). A probe consists of two main components: a reactive group to label reduced Cys residues in proteins coupled with an alkyne group for tagging click chemistry (Figure 4C). The TEV-biotin tag contains three different components: a biotin group for streptavidin affinity purification, a TEV (Tobacco etch virus) protease recognition domain (ENLYFQG) conjugated to an isotopically labeled Valine (heavy or light), and an azide handle for click chemistry, and a linker between a reporter and the reactive group (Weerapana et al., 2007; Chen et al., 2017) (Figure 7). The mass shift between light and heavy version tags is 6.014 Da (Weerapana et al., 2010).

A typical workflow of the isoTOP-ABPP approach employs two different (light and heavy) isotopically labeled cleavable biotin-azide tags that are reacted with IA-alkyne probes. Copper(I)-catalyzed azide-alkyne cycloaddition (CuAAC) is used to label two different experimental Cys-proteomes (Figure 6A). Reactive cysteines in proteins from two distinct proteomes are firstly alkylated with IA-alkyne probe, subsequently conjugated with isotopically differentiated cleavable biotin-azide tag using CuAAC (Kolb et al., 2001). IA-labeled proteins from two different proteomes are combined and subjected to affinity purification on streptavidin beads, followed by on-bead tryptic digestion. All labeled cysteine-containing peptides are then released by sodium dithionite before analysis by LC-MS/MS (Figure 6A). The calculated heavy/light ratio of each labeled Cys peptide, obtained from MS1 level analysis, will reflect Cys reactivity changes between two proteomes.

Many attempts have been performed to improve the performance of isoTOP-ABPP, mainly focused on cysteine reactive electrophile probes, cleavable biotin-azide tags, and heavy isotope incorporations (Maurais and Weerapana, 2019).

**FIGURE 8:**
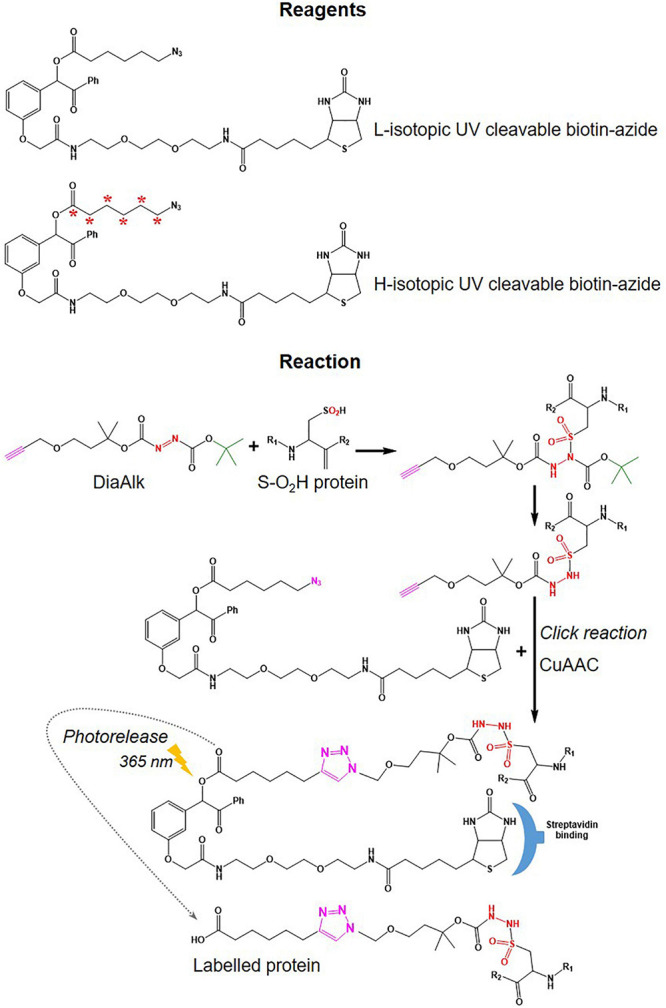
Tags used in DiaAlk workflows. Structure of UV-biotin tags used in DiaAlk and their reaction with oxidized Cys (*S*-0_2_H), purification using streptavidin, and release of labeled proteins by UV light.

• Various probes recently introduced (summarized in Figure 5C) include (i) IA-alkyne based probes such as chloropyridine (RB2) and fluoronitrobenzene (ERW3) (Shannon et al., 2014), a photocaged bromomethyl ketone (CBK) (Abo and Weerapana, 2015), iodomethyl ketone (CIK4), ethynyl benziodoxolone (EBX) (Abo et al., 2017), and (ii) light and heavy isotopic IA-alkyne tags (Abo et al., 2018), (iii) thiol-reactive probe (*N*-propargylmaleimide, NPM) (McConnell et al., 2020).
• Different cleavable biotin-azide tags: protease-cleavable biotin-azide tags for CuAAC-mediated conjugation (TEV protease), chemically cleavable azobenzene (Azo) linkers (Qian and Weerapana, 2017) (Figure 7), and photocleavable biotin-azide tags such as reductive dimethyl labeling (termed rdTOP-ABPP for triplex quantification) (Yang et al., 2018) and multiplexed thiol reactivity profiling (MTRP) (Tian et al., 2017), maleimide-activated (Figure 8).

• Heavy isotope incorporation: for duplex analysis including stable isotope labeling by amino acids in cell culture (SILAC), isotopes of IA-alkyne tags (Abo et al., 2018) (Figure 5C), light and heavy isotopic biotin-azide linkers (Figure 7); for triplex analysis including reductive dimethylation rdTOP-ABPP (Yang et al., 2018); and multiplex including isobaric tags (iTRAQ, TMT).

The synthesis of isotope containing cleavable biotin tags [L-Valine-N-Fmoc (^13^C_5_, ^15^N_1_)] is usually expensive and low yield, but recently, a more cost-effective version of light and heavy isotopes of IA-alkyne tags (Figure 5C) has been developed (Abo et al., 2018). The availability of these three crucial developments offers many combinations to tailor isoTOP-ABPP workflow to suit specific experimental designs. Furthermore, when combined with other MS-based approaches (next Section), different variants of isoTOP-ABPP can be customized to maximize Oxi-Cys detection and quantitation.

#### Quantitative Thiol Reactivity Profiling (QTRP)

In a strategy similar to isoTOP-ABPP, a quantitative thiol reactivity profiling (QTRP) approach was introduced for Oxi-proteomics analysis in which IAM-alkyne probes are used together with click chemistry for light and heavy isotope labeling (Fu et al., 2017). This approach also uses an electrophilic IAM-alkyne probe (2-iodo-*N*-(prop-2-yn-1-yl)acetamide, IPM, Figure 5C) coupled with isotopically light (^12^C) and heavy (^13^C) UV-cleavable azido-biotin tags via a click chemistry reaction (CuAAC) (Figure 8). All free reduced Cys thiol groups are firstly alkylated with IPM while reversible Cys are reduced by DTT, alkylated with IAM and then digested into peptides (Figure 6B). The peptides containing the IPM probe, from each sample, are then conjugated with light or heavy UV-cleavable azido-biotin tags (for control and compared samples) *via* copper catalyzed alkyne azide cycloaddition reaction (CuAAC, click chemistry) (Fu et al., 2017). Equal amounts of each sample (light or heavy labeled peptides) are combined and purified by streptavidin. Isotopically labeled peptides are then released *via* a photorelease step (irradiated with 365 nm UV light) (Fu et al., 2017). Mixed labeled peptides are then analyzed by MS, and the light/heavy ratio of a given precursor peptide derived from all isotopically labeled Cys peptides will indicate the oxidation level of control against experimental samples. The key difference between isoTOP-ABPP and QTRP is the order of the labeling steps; while a chemistry click reaction is performed at the protein level in isoTOP-ABPP approach, its reaction is carried out at the peptide level in the QTRP approach, offering a higher yield of biotinylated peptides (Fu et al., 2017), and high selectivity. Recently an updated QTRP approach has been introduced and enabled to detect 5,000 unique Cys-containing peptides (Fu et al., 2020).

#### Electrophilic Diazene Probe (DiaAlk)

There is controversy regarding the biological functions of *S*-sulfinylation and the extent of its reversibility. Furthermore, the analysis of *S*-sulfinylation is challenged by other canonical forms of Oxi-Cys, such as *S*-sulfhydration (-SSH). Indeed, a mass difference of 32 Da between unmodified and *S*-sulfinylated proteins in a standard proteomics workflow has been used to detect *S*-sulfinic acid by MS (*S*-O_2_H). However, this type of analysis has faced challenges, for example, the problem of oxidation during sample preparation, the interference of other PTMs with the same nominal mass shift [*S*-sulfhydration (-SSH), for example] (Lee et al., 2013). To overcome these problems, an alkyne derivative of di-t-butyl azodicarboxylate (DBAD) (termed DiaAlk), coupled with isotopic tags, has been used (Akter et al., 2018). The addition of the single alkyne tag to convert DBAD into an asymmetrical molecule enables sulfinic acid to be tagged at both positions of the N = N link in DiaAlk (Akter et al., 2018) (Figure 8). Reduced Cys residues in proteins (in both control and experimental samples) are blocked with 4,4′-dipyridyldisulfide (4-DPS) to prevent thiol-mediated probe consumption whilst sulfinylated Cys residues are reacted with DiaAlk probe (Figure 6C). Subsequently, other remaining types of Oxi-Cys residues are reduced (by DTT) and alkylated (by IAM) to prevent the re-oxidation of newly formed reduced Cys. Proteins are enzymatically digested (with trypsin), and *S*-sulfinylated peptides are then conjugated to light and heavy azide-biotin to form a photocleavable linker (Az-UV-biotin) *via* Cu(I)-catalyzed azide-alkyne cycloaddition (Figure 8) (for two distinct proteomes). Peptides from control and experimental samples are combined before being purified using streptavidin resin. Labeled-biotinylated peptides are released by photocleavage of the biotin linker by irradiation under UV light at 395 nm (Akter et al., 2018) and analyzed by LC-MS/MS.

## Enrichment of Oxi-Cys PTMs

Given the content of Cys in proteins is low and oxidized Cys is present at even lower levels, Cys enrichment is an important step to increase the coverage of Oxi-Cys by MS. There are three main methods to enrich Cys [details see Gu and Robinson (2016b)] as listed below:

• Biotin switch technique (BST): ICAT, iTRAQ, SILAC.
• Thiol-affinity RAC: OxcysDML, OxcyscPILOT.
• Immunoaffinity purification: iodoTMT, light and heavy isotopes NEM.

**FIGURE 9:**
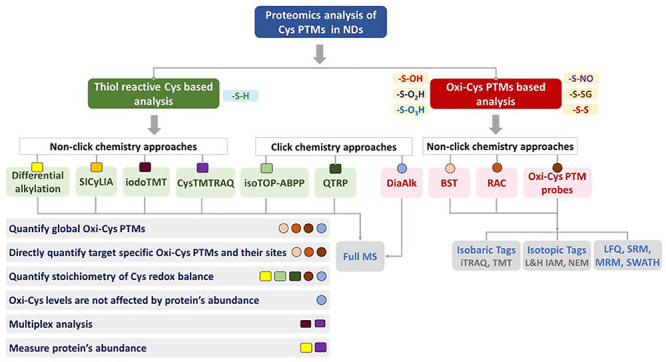
Considerations for choosing strategies for proteomic analysis of cysteine oxidation. Various features of each approach are included to help guide the choice of methods. The shape (□,○) and color code each represent a distinct method but are grouped according to the different overall approaches.

When Oxi-Cys quantitative analysis is performed across multiple samples, it is required that the level of Cys oxidation needs to be normalized to the level of protein expression, usually requiring a separate experiment for global proteome quantitation. However, approaches have been developed to generate Cys oxidation ratios and measure protein expression in the same experiment. In a differential alkylation approach, peptides containing Cys (either chemically alkylated or initially oxidized) are used to estimate Cys oxidation levels, while peptides not containing Cys are used for measurement of protein expression. Likewise, in cysTMTRAQ, differential isotope tagging of Cys-containing and peptides that do not contain Cys allow for measurement of both Cys oxidation and protein expression. Therefore, if access to MS instrument time is limited, choosing an approach that generates oxidation and protein expression data is worth considering (Figure 9).

### Biotin Switch Technique (BST)

The BST technique exploits the specificity and strong binding affinity of biotin with avidin resin to enrich biotinylated Cys-containing peptides from complex samples. Many approaches such as iodoTMT, CysTMTRAQ, QTRP, TOP-ABPP use BST to enrich Cys-containing peptides/proteins. This is an important step in these approaches to enhance the abundance and coverage of Oxi-Cys in biological samples. BST is widely used as a robust technique to enrich Cys; however, non-specific interactions might also occur if wash steps are not sufficiently stringent and bound peptides/proteins are challenging to elute if cleavable biotin is not used. In addition, the size of the biotin tags could also prevent appropriate interactions with the Cys residue (Gu and Robinson, 2016b).

Jaffrey et al. (2001) used BST to investigate *S*-nitrosylated proteins. All Cys-containing free thiols are alkylated with MMTS, while nitrosothiols are selectively reduced with ascorbate to form free thiols, which are then blocked with thiol-modifying reagent biotin–HPDP (*N*-[6-(biotinamido) hexyl]-3′-(2′-pyridyldithio) propionamide). The biotinylated proteins are then purified using streptavidin-agarose resin and selectively eluted with 2-mercaptoethanol (2-ME) to break the disulphide linkage in biotin-HPDP. However, cysteine residues modified by MMTS and endogenous disulphides are also reduced to form free thiol during this elution step (Jaffrey et al., 2001). Therefore, other alkylating reagents such as IAM or NEM should be used to alkylate Cys irreversibly to avoid this problem. Proteins are separated on a Sodium dodecyl sulfate –polyacrylamide-gel electrophoresis (SDS–PAGE), selected bands are then subjected to tryptic digestion and MS analysis for identification of *S*-NO PTMs (Jaffrey et al., 2001).

Another BST-based approach, *SNO*TRAP (*S*-NO trapping by triaryl phosphine) was developed to selectively target *S*-nitrosylated proteins to reveal those involved in the early stages of neurodegeneration in a mouse model of Alzheimer’s disease (Seneviratne et al., 2016). All initially reduced Cys are blocked with IAM while *S*-nitrosylated proteins are directly reacted with a biotin-*SNO*TRAP (*b*-SNOTRAP) probe to yield an azaylide intermediate to form the disulfide–iminophosphorane compound, which then is subject to reduction by TCEP before blocking with NEM (Seneviratne et al., 2016). The biotin-based enrichment of *S*-NO is performed at either protein or peptide levels.

### Thiol-Affinity Resin-Assisted Capture (RAC)

The enrichment of Cys-containing peptides/proteins based on thiol-affinity RAC has been widely used in quantitative proteomics analysis of reversible Oxi-Cys. Coupled with different quantitative proteomics approaches (e.g., isotopic labeling, isobaric tags, and LFQ), thiol-affinity resin has been used for the purification of various kinds of selected Oxi-Cys PTMs, including *S*-NO [SNO-RAC coupled with iTRAQ (Forrester et al., 2009), OxcyscPILOT/SNO-RAC coupled with TMT (Gu and Robinson, 2016a), *S*-SG (Guo et al., 2014), and global Oxi-Cys PTMs RAC coupled with LFQ (Paulech et al., 2013)]. The most popular RAC is Thiopropyl sepharose^®^ 6B resin that consists of a chemically stable ether linkage formed by an interaction between a reactive 2-thiopyridyl disulfide group with sepharose (Supplementary Figure 2). RAC is used for the enrichment of Cys-containing peptides through the formation of a covalent disulfide bond between the Cys and the thiopropyl group of the resin. Non-Cys-containing peptides are washed away by washing buffer (see Guo et al., 2014 for details), and Cys-containing peptides are eluted using a reducing reagent (DTT). Eluted peptides can be directly analyzed by LC-MS/MS with label-free quantification or can be labeled with either stable isotopic labeling or isobaric tags if higher quantitative accuracy is required.

## Quantitative MS-Based Approaches for Analysis of Oxi-Cys PTMs

There are two main types of data acquisition strategies, data-dependent acquisition (DDA) and data-independent acquisition (DIA). In DDA mode, a fixed number (Top N approach) of peptides are selected based on their intensity in MS1 scans, for fragmentation in MS2 scans. MS1 (intact peptide mass) and MS2 (fragment ion masses) data are analyzed using database-searching algorithms to score peptide-spectral matches (PSMs) for peptide/protein identification. Peptides are usually quantified using either MS1 or MS2 data, and well-established statistical and bioinformatics strategies are used to identify significantly different levels of peptides/proteins between conditions.

Many approaches use DDA for Oxi-Cys analysis, such as differential alkylation, iodoTMT, SICyCLIA *etc.*, but missing values across multiplex/multiple samples is an issue that can affect the statistical analysis of quantitative data. To overcome this, an unbiased technology in quantitative proteomics – DIA, such as sequential window acquisition of all theoretical mass spectra (SWATH-MS) and targeted DIA (SRM/MRM), can be used (Gillet et al., 2012). In a typical DIA analysis, a relatively wide mass/charge window is used for the isolation of peptides for fragmentation. In doing so, all detectible peptides are fragmented and quantified, which results in improved accuracy and precision of quantitation at the protein level with fewer missing values. The complexity of MS2 data in DIA, however, results in a challenge for data analysis, as it requires more sophisticated computation compared to DDA to identify peptides from spectra generated by the fragmentation of several peptides in a single MS2 scan. Approaches, where specific proteins/peptide are targeted for analysis (SRM/MRM and PRM) are also widely used for a robust quantitation of selected peptides/proteins and their PTMs.

### Label-Free Quantification (LFQ)

The development of advanced MS instrumentation (with high mass accuracy, resolution and sensitivity), together with highly reproducible nanoflow chromatography, has allowed the widespread use of LFQ-based quantification in bottom-up proteomics. LFQ can be used instead of labeling approaches (stable isotopic labeling or isobaric tags) to quantify proteins/peptides using sequential LC-MS/MS analysis of samples. Compared to labeling approaches, LFQ offers a higher dynamic range than isotope labeling methods, but the quantitative accuracy is lower, and typically, more replicates are required for robust quantitation. In this approach, each digested proteome is run separately on an LC-MS/MS platform, and the quantitation is carried out based on XIC (extracted ion chromatogram) intensities (Wang et al., 2008). In XIC, the ion current signal (in MS1) associated with each individual peptide is used to reconstruct the area under the curve during chromatography which is proportional to the amount of peptide present. The performance of LFQ-based quantification can be improved by the incorporation of more advanced data processing. For example, retention time alignment across samples and “matching between runs” in which missing values are reduced by transferring peptide identifications based on narrow mass and retention time windows. This minimizes missing values due to poor fragmentation data or cases where a peptide is not selected for fragmentation because it was present at a lower amount in one sample compared to others in an experiment.

### Stable-Isotope Dimethyl Labeling

Stable-isotope dimethyl labeling employs different isotopomers of formaldehyde and cyanoborohydride to introduce dimethyl groups to the N-termini and side chains of lysine residues which differ by at least 4 Da. This is used to label usually 2–3 samples, but up to 5 samples (5-plex) can be labeled and pooled in a single experiment if other formaldehyde/cyanoborohydride isotopomers are used (Wu et al., 2014). The application of multiplexing for labeling multiple samples in a single experiment offers many benefits as analysis of multiple samples can be performed in the same experiment and thereby reduced the amount of instrument time required. On the one hand, given that samples are pooled and analyzed within an LC-MS/MS acquisition, this type of quantification is more accurate than sequential label-free quantification approaches. However, because the samples are pooled, the peptide complexity is increased, and up-front peptide fractionation may be required depending on the initial sample complexity and acquisition speed of the MS instrument.

### Multiplex Isobaric Tagging

Multiplex approaches (iodoTMT and cysTMTRAQ) allow the analysis of up to six different samples in a single experiment. This is achieved by labeling samples with chemical tags which add the same overall mass to each peptide (same mass addition in MS1), but when labeled peptides are fragmented in MS2 scans, they generate signature (barcoded) ions used for quantitation of each individual sample. The key difference between isobaric tags and isotopic labeling approaches is that all isobaric tags are identical in total mass (see Figure 4C). In contrast, isotopic labeling masses are different when labeling peptides and peptides are distinguished by a mass difference in MS1 scans. The quantitation of peptides using isotopic labeling is performed based on the intensity of peptides measured in MS1 spectra, while that of the isobaric tagging approach is carried out based on the intensity of tags cleaved from labeled peptides in MS2 spectra with signature masses (e.g., 126, 127, 128, 129, 130, and 131 m/z). In a complex digested sample, co-elution (from online-HPLC) and co-isolation (at MS1) of peptides with others with a similar m/z result can result in a combination of the reporter ion intensity from different peptides, resulting in a bias in the relative ratio of a peptide from different proteomes. This is a particular challenge for quantifying low abundance peptides/proteins as the interference is much higher. Specific data acquisition strategies and statistical analysis packages have been developed to address this issue of ratio compression (Pappireddi et al., 2019).

### Multiple Reaction Monitoring (MRM)

MRM (also known as SRM, Selected Reaction Monitoring) is used to analyze specific proteins/peptides of interest in a complex sample by selectively targeting peptides to enhance the reproducibility and accuracy of quantitation, especially for low abundance proteins/peptides. This approach involves the isolation of targeted peptides (enzymatically digested from proteins of interest) and specific fragment ions from the targeted peptides. This is typically performed in a triple quadrupole mass spectrometer. The first and third quadrupoles are used as filters to specifically select the targeted ions (m/z) corresponding to peptides of interest and specific fragment ions fragmented from the targeted peptides. The second quadrupole acts as a collision cell for fragmentation of the targeted ions. The transition of ion pairs (precursors/fragment ions) are monitored continuously during the MS run to generate a spectrum that contains signal intensities of specific transitions as a function of time (Lange et al., 2008). Given that this approach uses a selective ion pair technique, not a “full scan” technique like others, it can improve sensitivity by up to two orders of magnitude and the linear response over a wide dynamic range up to five orders of magnitude compared to “full scan” technique (Lange et al., 2008). MRM can measure peptides from more than 100 proteins per acquisition using high-speed triple quadrupole MS in line with accurate retention time scheduling (Percy et al., 2014). A variant of this approach, Parallel-reaction monitoring (PRM), can be performed using a high-resolution instrument such as the Orbitrap instrument. Unlike MRM in which typically three fragment ions are monitored in transitions, in PRM all fragment ions are measured for the set of targeted peptides (Meyer and Schilling, 2017).

An approach named OxiMRM has been developed for targeted Oxi-Cys proteomics analysis. It utilizes a stable isotopic approach to label Cys residues *via* two different alkylating steps, light (d_0_) and heavy (d_5_) stable isotope labeled NEM to label reduced and Oxi-Cys, respectively (Figure 4B). These labeled proteins are then subjected to immunoaffinity purification to enrich a specific protein of interest before being digested with trypsin and quantified using a multiple reaction monitoring (MRM) method (Held et al., 2010). This technique offers a highly sensitive and reproducible approach to target specific Cys residues in proteins of interest that can be used to quantify both sulfinic and sulfonic acid oxidation levels (Held et al., 2010). Another method in this category, UPLC-pSRM (combination of nano-UPLC and pseudo-SRM performed on a LTQ-Orbitrap), has been introduced to detect and quantify irreversible Cys oxidation (Lee et al., 2013) without any enrichment step. The approach employs a high chromatographic resolution (using a long reverse-phase nano-UPLC column) to separate different forms of Oxi-Cys and a high mass accuracy, resolution and sensitivity of an Orbitrap instrument for identification and quantitation of modified Cys sites (Lee et al., 2013). The benefit of this technique is that it allows direct detection of irreversibly linked Oxi-Cys residues in peptides/proteins.

### Cysteine-Specific Data Independent Analysis (Cys-DIA)

The ultimate benefit of DIA is that it can measure most detectible peptides falling in a selected *m/z* window to improve the accuracy and precision of peptide quantitation with minimal missing values. In that context, Cys-DIA was used to analyze Cys-containing peptides purified with Thiopropyl Sepharose^TM^ 6B beads (as discussed in section “Thiol-affinity resin-assisted capture”) to increase Cys-proteome coverage up to 35% compared to conventional DIA/SWATH approach. In brief, all reversible Oxi-Cys residues are reduced by (DTT or TECP), and all free reduced Cys residues (newly formed and native reduced Cys) are subjected to Cys purification. These purified Cys-peptides are then analyzed on an Orbitrap instrument (Q-Exactive HF-X) operated in high-resolution MS1 DIA mode (HRMS1-DIA) to quantify Cys-containing peptides (Tahir et al., 2020). A typical selection of 25–50 *m/z* windows covering from 400 to 1500 *m/z* is used, and all ionized peptides within that range are fragmented, generating complex spectra of fragmented ions. Unlike DDA data, DIA data can be searched against a peptide spectral reference library generated from separate DDA experiments to identify and quantitate peptides (Tahir et al., 2020).

## Cysteine Oxidation in Neurodegenerative Diseases

We have surveyed published articles using PubMed^1^, from the last 20 years, for studies that include proteomic analysis of Cys oxidation in neurodegenerative diseases with different keywords, as shown in Figure S1. Keywords for searching included: proteo^∗^ and oxida^∗^ and cysteine (used as fixed keywords), and then other variables such as neuro^∗^, human, AD, Parkinson disease (PD), multiple sclerosis (MS), and motor neuron disease (MND)/ALS. Generally, Oxy-Cys studies have steadily increased over the last two decades. However, when specific neurodegenerative disease keywords (AD, PD, MS, MND, and ALS) were included, the number of proteomics studies of Cys oxidation in the context of neurodegenerative disease is strikingly low. Many hits were found for PD and AD, but when these hits were manually inspected, the total number of studies for Oxi-Cys proteomics was less than ten; especially a combination of fixed keywords with human and MND (or ALS) resulted in no hits.

Moreover, we have also examined data from the CSF (cerebrospinal fluid) Proteome Resource^2^ containing 128 human CSF proteomics datasets (4,192 proteins) available from 31 research articles for neurodegenerative disease studies (AD, MS, PD, and ALS) from 2010 to 2019 (Guldbrandsen et al., 2017), there were no Oxy-Cys proteomic studies. This indicates that despite the oxidative state of Cys playing important roles in neurodegenerative diseases, very little (or no) studies have been performed to investigate how global changes in Cys oxidation are altered in neurodegenerative diseases or how it changes in response to disease progression and treatment. Here, we review recent Oxi-Cys PTMs studies to characterize oxidation changes in neurodegenerative diseases.

### Alzheimer’s Disease

*S*-NO is implicated in AD through its association with impaired metabolism, mitochondrial dysfunction, neuronal loss, and protein misfolding and aggregation (Dyer et al., 2017). Various methods have been used to measure changes in *S*-NO in mouse models of AD and clinical samples. Using the OxcyscPILOT approach, Dyer et al. (2017) identified 135 *S*-NO modified proteins, of which 11 proteins were differentially regulated in an AD (APP/PS1) mouse model. This approach allows comparison of *S*-NO levels across different samples (up to 6 samples) as well as measuring total (reduced and reversibly oxidized) cysteine-containing peptides so that the levels of *S*-NO can be easily normalized in a single experiment (Dyer et al., 2017). However, this technique has not yet been applied to the analysis of clinical samples.

Synaptic loss and neuroinflammation contribute to early pathophysiological changes in AD. The induction of nitric oxide synthase type 2 leads to increased nitric oxide levels and *S*-NO of cysteine residues (Paula et al., 2010). An isobaric tag approach, iodoTMT, was used to reveal the *S*-NO sites on Cys residues of NOS2-dependent *S*-NO of synaptic proteins in mild cognitive impairment (MCI) and AD and human control brain tissues (Wijasa et al., 2020). Of 291 and 295 *S*-NO proteins identified in synaptosome fractions, 16 and 9 *S*-NO proteins were significantly increased in AD vs. MCI brains and AD vs. Ctrl, respectively (Wijasa et al., 2020). Of these up-regulated *S*-NO proteins, three proteins, myelin-oligodendrocyte glycoprotein (MOG), glutathione *s*-transferase Mu 3 (GSTM3), and four and a half LIM domains protein 1 (FHL1), were found at a higher level in AD compared to either MCI or Ctrl cases (Wijasa et al., 2020). Furthermore, this study identified 23 potential *S*-NO biomarkers (see Wijasa et al., 2020 for details). The up-regulation of MOG was associated with an increased production of autoantibodies against MOG protein in AD, indicating early demyelination of the hippocampal region (Papuć et al., 2015). The exact functions of FHL1 or GSTM3 in synapses are still unclear (Wijasa et al., 2020). Other *S*-NO modified proteins such as neurocan core protein (NCAN), calmodulin-dependent protein kinase type II subunit gamma (CAMK2G), CD44 and aspartate aminotransferase (GOT1) were also reported to be increased in abundance in AD brains as well as in human CSF (Wijasa et al., 2020), representing potential AD biomarkers for future study.

Antibodies to measure specific Cys oxidation types have been used for the detection and measurement of protein oxidation in neurodegenerative diseases, e.g., *S*-nitrosocysteine antibody for *S*-NO. In an early Oxi-Cys proteomics study, Choi et al. (2004) applied a 2-D protein gel-based approach coupled with immunostaining and MS analysis to discover three human brain UCH-L1 isoforms that contain Oxi-Cys, suggesting the oxidation might damage the ubiquitination/de-ubiquitination machinery involved in the etiology of AD and PD. Newman et al. (2007) utilized 2-D-Oxyblots and MS analysis to identify specific targets of *S*-glutathionylated Cys in AD inferior parietal lobule (IPL) compared to control IPL, in which three proteins, deoxyhemoglobin, α-crystallin B, glyceraldehyde phosphate dehydrogenase (GAPDH), and α-enolase, were significantly *S*-glutathionylated in AD IPL (Newman et al., 2007). The exact functions of these *S*-glutathionylated proteins were not elucidated, but the study identified several proteins sensitive to oxidative stress in the AD brain (Newman et al., 2007). Using a similar approach, Zahid et al. (2014) reported three proteins [voltage-dependent anion-selective channel protein 2 (VDAC2), superoxide dismutase [Mn] (SOD2), and fructose-bisphosphate aldolase C (ALDOC)] that were *S*-glutathionylated in autopsied brain specimens (hippocampus, substantia nigra and cortex) (Zahid et al., 2014). Although MS was used to identify *S*-glutathionylated proteins, the exact location of Oxi-Cys sites within these proteins was only predicted by *in silico* analysis. The altered levels of *S*-NO in AD might contribute to disease-related consequences (Zahid et al., 2014).

Transthyretin (TTR), an abundant CSF protein, contains both free and oxidized Cys residues. The relative changes between these two states generate different TTR isoforms under oxidative stress conditions (Poulsen et al., 2014). This protein can bind to beta-amyloid peptide, and familial amyloidosis diseases (e.g., familial amyloid polyneuropathy) have been associated with its variants (Biroccio et al., 2006). In a later study, Poulsen et al. (2014) adopted this approach to characterize the oxidation isoforms of TTR in CSF from AD, MCI, and normal pressure hydrocephalus (NPH) patients and HCs, and an increased oxidation level of Cys_10_ in TTR isoforms was observed in AD and MCI compared to other groups. This offers a potential novel biomarker for the diagnosis of AD in patients but would need to be investigated in larger cohorts of patients for validation.

### Multiple Sclerosis

Multiple sclerosis is a neuroinflammatory disease associated with demyelination and axonal damage (Compston and Coles, 2002). Oxidative stress is a crucial factor contributing to the disease pathology *via* demyelination and oligodendroglia degeneration (Konat and Wiggins, 1985). Using a modified proteomics approach, which excluding reduction and alkylation steps to retain glutathione attached to cysteine through disulfide linkage, coupled with a targeted database search, Srivastava et al. (2019) characterized *S*-glutathionylation of CSF proteins in MS. In particular, they identified several proteins that were glutathionylated in patients with MS during a relapse, including three proteins (ECSOD, A1AT, and PLTP) which were oxidized at functionally important positions in their structure (Srivastava et al., 2019). This indicates that *S*-glutathionylation may negatively regulate the function of these proteins in MS (Liedhegner et al., 2012). However, given that this study has a limited sample size, validation of these oxidation changes needs to be performed in larger patient cohorts to confirm a link between *S*-glutathionylation and MS (Srivastava et al., 2019).

## Discussion and Future Directions

Over the last 20 years, proteomic analysis of Cys oxidation has revealed global profiles of the redox network in many biological systems. Two main types of analyses have been performed, oxidation site mapping and quantitation and thiol reactivity profiling (Fu et al., 2020). The site mapping Oxi-Cys strategy has many approaches (BST, RAC, iodoTMT, *etc.*) to identify redox-sensitive cysteines in proteins and quantitation of dynamic changes in cysteine oxidation in response to a variety of perturbations (Jaffrey et al., 2001; Forrester et al., 2009; Guo et al., 2014; Gu and Robinson, 2016a). A summary of approaches used for the analysis of Cys PTMs and their applications is shown in Figure 9, and further details of the advantages and disadvantages of each method are listed in Supplementary Table 1.

### Advantages and Disadvantages

The identification and quantitation of various forms of cysteine oxidation remain challenging because of the low abundance of cysteine in the proteome and the sub-stoichiometric nature of oxidation. It requires robust tools to characterize the Oxi-Cys proteome. As discussed above, many approaches, mostly attempting to enrich Oxi-Cys residues or Cys-containing peptides, have been established, such as iodoTMT, isoTOP-ABPP, QTRP, and DiaAlk. Selective Oxi-Cys proteomics approaches, mainly based on BST or probes, offer higher sensitivity, specificity and precision (Mnatsakanyan et al., 2019) than methods that don’t incorporate an enrichment step (differential alkylation, SICyLIA, or CysTMTRAQ). However, Oxi-Cys approaches, which rely on an enrichment step, might not directly capture the native oxidized state of proteins under physiological conditions. An alternative to this is the SICyLIA approach, a simple, unbiased and robust method to sensitively detect and accurately quantify global oxidized Cys-proteomes since it does not require Cys enrichment (van der Reest et al., 2018). However, given the lack of an enrichment step in the SICyLIA approach, it may be less suitable for the analysis of low abundance proteins and cases where the stoichiometry of oxidation is low.

Most of the approaches listed in Figure 9 (apart from Differential alkylation and DiaAlk) require an extra experiment/additional MS time to measure protein expression levels which is necessary for the normalization of Cys oxidation levels. This is an important consideration if protein expression differences are likely to occur between experimental and control conditions.

Most of the methods shown in Figure 9 are not directly compatible with the analysis of irreversible cysteine oxidation. However, in some cases, data from some approaches such as differential alkylation or SICyLIA can allow the identification of irreversible Cys oxidation (*S*-O_3_H or *S*-O_2_H) if these modifications are selected in database searching algorithms. Click chemistry-based approaches offer many benefits for the study of reversible Oxi-Cys residues, such as high selectivity and direct measurement and determination of exact sites of Cys containing PTMs. However, there are no available probes designed for targeting and purifying peptides/proteins containing irreversible Oxi-Cys (*S*-O_3_H), but a few for *S*-O_2_H, such as the DiaAlk probe have been developed (Figure 5B).

Isobaric tag-based approaches (iodoTMT and CysTMTRAQ) have been used in several Oxy-Cys PTM studies, redox and electrophile reactive Cys proteome applications (Gygi et al., 1999; Jaffrey et al., 2001; Chouchani et al., 2017) because of the commercial availability of reagents as well as their multiplexing capability. However, the high cost of reagents and high-end MS instrumentation required to avoid ratio compression due to co-isolated peptides is potentially a barrier to the wide adoption of these approaches. Furthermore, the large structure of isobaric tag labeling reagents may have reduced accessibility to react with sterically buried Cys in native conditions (Fu et al., 2020).

### Perspectives

Recent advances in MS-based proteomics, biomarker discovery in the context of neurodegenerative disease has significant potential. Applications include the identification of biomarkers for earlier diagnosis of disease and biomarkers of drug-target engagement that can be used for the development and testing of new therapies.

Recent studies using protein biomarkers for AD diagnosis highlight the advances in biomarker discovery for neurodegenerative diseases. The diagnosis of AD relies on a combination of clinical criteria, brain scans and a neurological exam. Disease pathology is reflected in biomarkers, including amyloid-beta 1–42 peptide (Aβ_1__–__42_), tau protein (t-tau), and tau phosphorylated at Thr-181 (p-tau181) in CSF (Begcevic et al., 2018). However, only two validated methods have been used to identify amyloid-beta in the brain; measurement of levels in CSF or positron-emission tomography (PET) brain imaging (Nakamura et al., 2018). Nakamura et al. (2018) recently developed an immunoprecipitation-MS (IP-MS) based assay to measure amyloid-beta precursor protein (APP)_669__–__711_/amyloid-β (Aβ)_1__–__42_ and Aβ_1__–__40_/Aβ_1__–__42_ ratios in plasma to predict brain amyloid-β burden. The performance of this composite biomarker was high [areas under the receiver operating characteristic curves (AUCs) in both data sets (discovery, 96.7%, *n* = 121 and validation, 94.1%, *n* = 111)]. It had an accuracy of 90% compared to diagnosis with PET imaging, demonstrating the potential clinical utility of this MS-based biomarker assay (Nakamura et al., 2018).

Going beyond protein expression level biomarkers to protein modification markers in neurodegenerative disease is the next step toward accessing, for example, earlier markers of disease progression and a means to evaluate therapeutic efficacy. For example, the application of Oxi-Cys proteomics approaches to study changes in oxidation in CSF from patients in longitudinal studies may reveal proteins with an oxidation status that correlates to disease progression. The efficacy of therapeutics designed to reduce oxidative stress in patients could be tested for their ability to reduce the level of these oxidation biomarkers and would therefore allow measurement of target engagement in clinical studies. There is also a variety of preclinical applications of Oxi-Cys proteomics in which the impact of specific human disease mutations on oxidation profiles can be assessed in animal models.

It is necessary to develop reliable proteomics approaches to build a global resource of (reversibly and irreversibly) Oxi-Cys proteomes in different clinical samples and types of neurodegenerative diseases (brain tissues, CSF, blood serum, *etc.*) that can be used to identify biomarkers in diagnosis and drug development studies. This resource could focus on: specific cell types of global Oxi-Cys proteomes to understand the disease mechanism of neurodegenerative diseases; integration of Oxi-Cys proteomics networks in brain tissue, CSF or plasma. Furthermore, these will also contribute to the understanding of neurodegenerative disease pathogenesis, progression, monitoring and treatment. Indeed, the integration of Oxi-proteomics data and data from the metabolomics of oxidative stress combined with *in silico* computational models, will provide a clearer picture of oxidative stress processes in neurodegenerative diseases.

## Author Contributions

TP, WB, and RM wrote the first draft of the manuscript. PS and MC edited the manuscript. All authors contributed to the article and approved the submitted version.

## Conflict of Interest

The authors declare that the research was conducted in the absence of any commercial or financial relationships that could be construed as a potential conflict of interest.

## Footnotes

1 https://pubmed.ncbi.nlm.nih.gov/

2 https://proteomics.uib.no/csf-pr/

## Abbreviations

2-D gel: two-dimensional gel
2-ME: 2-mercaptoethanol
AD: Alzheimer’s disease
ALS: amyotrophic lateral sclerosis
A β: amyloid-beta
Biotin–HPDP: *N*-[6-(biotinamido) hexyl]-3 ′ -(2 ′ -pyridyldithio) propionamide
BME: β-mercaptoethanol
BST: biotin switch technique
CAM: carbamidomethyl group
CBK: photocaged bromomethyl ketone
CIK4: iodomethyl
CNS: central nervous system
CSF: cerebrospinal fluid
Ctrl: control
CuAAC: copper(I)-catalyzed azide–alkyne cycloaddition
Cys: cysteine
CysTMT: cysteine-reactive tandem mass tag
CysTMTRAQ: cysteine-reactive tandem mass tag and isobaric tags for relative and absolute quantitation
DDA: data-dependent acquisition
DIA: data-independent acquisition
DTBA: dithiobutylamine
DTT: dithiothreitol
EBX: ethynyl benziodoxolone
ELISA: enzyme-linked immunosorbent assay
ERW3: fluoronitrobenzene
ESI: electrospray ionization
FFI: fatal familial insomnia
FTD: frontotemporal dementia
HC: healthy control
HPDP: *N*-[6-(biotinamido) hexyl]-3 ′ -(2 ′ -pyridyldithio) propionamide (biotin–HPDP)
HRMS1: high resolution MS1
IA: immunoaffinity
IAA: iodoacetic acid
IAM: iodoacetamide
iCAT: isotope-coded affinity tag
iodoTMT: iodoacetyl isobaric tandem mass tags
IPL: inferior parietal lobule
isoTOP-ABPP: isotopic tandem orthogonal proteolysis-activity-based protein profiling
iTRAQ: isobaric tags for relative and absolute quantitation
LFQ: label free quantification
MCI: mild cognitive impairment
MMTS: methanethiosulfonate
MND: motor neuron disease
MnSOD: manganese superoxide dismutase
MRM: multiple reaction monitoring
MS: mass spectrometry
MS: multiple sclerosis
MTRP: multiplexed thiol reactivity profiling
NaAsc: sodium ascorbate
ND: neurodegenerative diseases
NEM: *N*-ethylmaleimide
NLRP3, NOD-like receptor family: pyrin domain containing 3
NMDA: *N*-methyl -D-aspartate
NOD: nucleotide-binding and oligomerization domain
NPH: normal pressure hydrocephalus
NPM: *N*-propargylmaleimide
OxcyscPILOT: oxidized cysteine-selective combined precursor isotopic labeling and isobaric tagging
Oxi-Cys: oxidative cysteine
PD: Parkinson’s disease
PRM: parallel reaction monitoring
p-SRM: pseudo- selected reaction monitoring
PTM: post-translational modification
RAC: resin-assisted capture
RB2: chloropyridine
ROS: reactive oxygen species
sCJD: Creutzfeldt–Jakob disease
SDS–PAGE: sodium dodecyl sulfate –polyacrylamide-gel electrophoresis
SH: sulfhydryl
SILAC: stable isotope labeling by amino acids in cell culture
*S*-NO: *S*-nitrosylation
*SNO*TRAP: *S*-NO trapping by triaryl phosphine
*S*-O _2_H: *S*-sulfinylation
*S*-O _3_H: *S*-sulfonylation
SOD1: superoxide dismutase type-1
*S*-OH: *S*-sulfenylation
SPS: synchronous precursor selection
SRM: selected reaction monitoring
*S*-SG: *S*-glutathionylation
SWATH-MS: sequential window acquisition of all theoretical mass spectra
TCA: trichloroacetic acid
TCEP: Tris(2-carboxyethyl)phosphine
TEV: tobacco etch virus
TMT: isobaric tandem mass tags
TOF: time-of-flight
TOP: tandem orthogonal proteolysis
TTP: transthyretin
UPLC: ultra performance liquid chromatography
XIC: extracted ion chromatogram.

## References

[B1] Abo M. Bak D. W. Weerapana E. (2017). Optimization of caged electrophiles for improved monitoring of cysteine reactivity in living cells. *Chembiochem* 18 81–84. 10.1002/cbic.201600524 27813293 PMC5209257

[B2] Abo M. Li C. Weerapana E. (2018). Isotopically-labeled iodoacetamide-alkyne probes for quantitative cysteine-reactivity profiling. *Mol. Pharm.* 15 743–749. 10.1021/acs.molpharmaceut.7b00832 29172527 PMC5841085

[B3] Abo M. Weerapana E. (2015). A caged electrophilic probe for global analysis of cysteine reactivity in living cells. *J. Am. Chem. Soc.* 137 7087–7090. 10.1021/jacs.5b04350 26020833

[B4] Akter S. Fu L. Jung Y. Conte M. L. Lawson J. R. Lowther W. T. (2018). Chemical proteomics reveals new targets of cysteine sulfinic acid reductase. *Nat. Chem. Biol.* 14 995–1004. 10.1038/s41589-018-0116-2 30177848 PMC6192846

[B5] Andersen J. K. (2004). Oxidative stress in neurodegeneration: cause or consequence? *Nat. Med.* 10 18–25. 10.1038/nrn1434 15298006

[B6] Ansong C. Wu S. Meng D. Liu X. Brewer H. M. Deatherage Kaiser B. L. (2013). Top-down proteomics reveals a unique protein S-thiolation switch in *Salmonella* Typhimurium in response to infection-like conditions. *Proc. Natl. Acad. Sci. U S A* 110 10153–10158. 10.1073/pnas.1221210110 23720318 PMC3690903

[B7] Auclair J. R. Salisbury J. P. Johnson J. L. Petsko G. A. Ringe D. Bosco D. A. (2014). Artifacts to avoid while taking advantage of top-down mass spectrometry based detection of protein S-thiolation. *Proteomics* 14 1152–1157. 10.1002/pmic.201300450 24634066 PMC4507715

[B8] Barber S. C. Shaw P. J. (2010). Oxidative stress in ALS: key role in motor neuron injury and therapeutic target. *Free Radic. Biol. Med.* 48 629–641. 10.1016/j.freeradbiomed.2009.11.018 19969067

[B9] Begcevic I. Brinc D. Brown M. Martinez-Morillo E. Goldhardt O. Grimmer T. (2018). Brain-related proteins as potential CSF biomarkers of Alzheimer’s disease: a targeted mass spectrometry approach. *J. Proteomics* 182 12–20. 10.1016/j.jprot.2018.04.027 29684683

[B10] Biroccio A. Del Boccio P. Panella M. Bernardini S. Di Ilio C. Gambi D. (2006). Differential post-translational modifications of transthyretin in Alzheimer’s disease: a study of the cerebral spinal fluid. *Proteomics* 6 2305–2313. 10.1002/pmic.200500285 16552785

[B11] Bomgarden R. D. Viner R. I Kuhn K. Pike I. Rogers J. C. (2012). Iodoacetyl tandem mass tags for cysteine peptide modification, enrichment and quantitation. *iHUPO*

[B12] Bosco D. A. Morfini G. Karabacak N. M. Song Y. Gros-Louis F. Pasinelli P. (2010). Wild-type and mutant SOD1 share an aberrant conformation and a common pathogenic pathway in ALS. *Nat. Neurosci.* 13 1396–1403. 10.1038/nn.2660 20953194 PMC2967729

[B13] Brosnan J. T. Brosnan M. E. (2006). The sulfur-containing amino acids: an overview. *J. Nutr.* 136 1636s–1640s. 16702333 10.1093/jn/136.6.1636S

[B14] Chen C.-H. Li W. Sultana R. You M.-H. Kondo A. Shahpasand K. (2015). Pin1 cysteine-113 oxidation inhibits its catalytic activity and cellular function in Alzheimer’s disease. *Neurobiol. Dis.* 76 13–23. 10.1016/j.nbd.2014.12.027 25576397 PMC4423621

[B15] Chen L.-N. Shi Q. Zhang B.-Y. Zhang X.-M. Wang J. Xiao K. (2016). Proteomic analyses for the global S-Nitrosylated proteins in the brain tissues of different human prion diseases. *Mol. Neurobiol.* 53 5079–5096. 10.1007/s12035-015-9440-7 26392294

[B16] Chen X. Wong Y. K. Wang J. Zhang J. Lee Y.-M. Shen H.-M. (2017). Target identification with quantitative activity based protein profiling (ABPP). *Proteomics* 17:1600212. 10.1002/pmic.201600212 27723264

[B17] Choi J. Levey A. I. Weintraub S. T. Rees H. D. Gearing M. Chin L. S. (2004). Oxidative modifications and down-regulation of ubiquitin carboxyl-terminal hydrolase L1 associated with idiopathic Parkinson’s and Alzheimer’s diseases. *J. Biol. Chem.* 279 13256–13264. 10.1074/jbc.m314124200 14722078

[B18] Chouchani E. T. James A. M. Methner C. Pell V. R. Prime T. A. Erickson B. K. (2017). Identification and quantification of protein S-nitrosation by nitrite in the mouse heart during ischemia. *J. Biol. Chem.* 292 14486–14495. 10.1074/jbc.m117.798744 28710281 PMC5582841

[B19] Compston A. Coles A. (2002). Multiple sclerosis. *Lancet* 359 1221–1231. 11955556 10.1016/S0140-6736(02)08220-X

[B20] Dalle-Donne I. Scaloni A. Giustarini D. Cavarra E. Tell G. Lungarella G. (2005). Proteins as biomarkers of oxidative/nitrosative stress in diseases: the contribution of redox proteomics. *Mass Spectrom Rev.* 24 55–99. 10.1002/mas.20006 15389864

[B21] Durham T. B. Blanco M. J. (2015). Target engagement in lead generation. *Bioorg. Med. Chem. Lett.* 25 998–1008. 10.1016/j.bmcl.2014.12.076 25630223

[B22] Dyer R. R. Gu L. Robinson R. A. S. (2017). “S-nitrosylation in Alzheimer’s disease using oxidized cysteine-selective cPILOT,” in *Current proteomic approaches applied to brain function*, eds Santamana E. Fernández-Irigoyen J. (New York: Springer), 225–241. 10.1007/978-1-4939-7119-0_14

[B23] Erickson B. K. Jedrychowski M. P. Mcalister G. C. Everley R. A. Kunz R. Gygi S. P. (2015). Evaluating multiplexed quantitative phosphopeptide analysis on a hybrid quadrupole mass filter/linear ion trap/orbitrap mass spectrometer. *Anal. Chem.* 87 1241–1249. 10.1021/ac503934f 25521595 PMC4303329

[B24] Fang J. Nakamura T. Cho D.-H. Gu Z. Lipton S. A. (2007). S-nitrosylation of peroxiredoxin 2 promotes oxidative stress-induced neuronal cell death in Parkinson’s disease. *Proc. Natl. Acad. Sci.* 104 18742–18747. 10.1073/pnas.0705904104 18003920 PMC2141847

[B25] Forrester M. T. Thompson J. W. Foster M. W. Nogueira L. Moseley M. A. Stamler J. S. (2009). Proteomic analysis of S-nitrosylation and denitrosylation by resin-assisted capture. *Nat. Biotechnol.* 27 557–559. 10.1038/nbt.1545 19483679 PMC2891235

[B26] Fu L. Li Z. Liu K. Tian C. He J. He J. (2020). A quantitative thiol reactivity profiling platform to analyze redox and electrophile reactive cysteine proteomes. *Nat. Protoc.* 15 2891–2919. 10.1038/s41596-020-0352-2 32690958

[B27] Fu L. Liu K. Sun M. Tian C. Sun R. Morales Betanzos C. (2017). Systematic and quantitative assessment of hydrogen peroxide reactivity with cysteines across human proteomes. *Mol. Cell. Proteomics* 16 1815–1828. 10.1074/mcp.ra117.000108 28827280 PMC5629266

[B28] Gillet L. C. Navarro P. Tate S. Röst H. Selevsek N. Reiter L. (2012). Targeted data extraction of the MS/MS spectra generated by data-independent acquisition: a new concept for consistent and accurate proteome analysis. *Mol. Cell. Proteomics* 11:16717. 10.1074/mcp.O111.016717 PMC3433915 22261725

[B29] Group W. G. E. M.-A. S. (2017). Safety and efficacy of edaravone in well defined patients with amyotrophic lateral sclerosis: a randomised, double-blind, placebo-controlled trial. *Lancet Neurol.* 16 505–512. 28522181 10.1016/S1474-4422(17)30115-1

[B30] Gu L. Robinson R. A. (2016a). High-throughput endogenous measurement of S-nitrosylation in Alzheimer’s disease using oxidized cysteine-selective cPILOT. *Analyst* 141 3904–3915. 10.1039/c6an00417b 27152368 PMC4904844

[B31] Gu L. Robinson R. A. (2016b). Proteomic approaches to quantify cysteine reversible modifications in aging and neurodegenerative diseases. *Proteomics Clin. Appl.* 10 1159–1177. 10.1002/prca.201600015 27666938 PMC5157156

[B32] Guldbrandsen A. Farag Y. Kroksveen A. C. Oveland E. Lereim R. R. Opsahl J. A. (2017). CSF-PR 2.0: an interactive literature guide to quantitative cerebrospinal fluid mass spectrometry data from neurodegenerative disorders. *Mol. Cell. Proteomics* 16 300–309. 10.1074/mcp.o116.064477 27890865 PMC5294216

[B33] Guo C. Sun L. Chen X. Zhang D. (2013). Oxidative stress, mitochondrial damage and neurodegenerative diseases. *Neural. Regen. Res.* 8 2003–2014. 25206509 10.3969/j.issn.1673-5374.2013.21.009 PMC4145906

[B34] Guo J. Gaffrey M. J. Su D. Liu T. Camp D. G. II Smith R. D. (2014). Resin-assisted enrichment of thiols as a general strategy for proteomic profiling of cysteine-based reversible modifications. *Nat. Protoc.* 9 64–75. 10.1038/nprot.2013.161 24336471 PMC4038159

[B35] Gygi S. P. Rist B. Gerber S. A. Turecek F. Gelb M. H. Aebersold R. (1999). Quantitative analysis of complex protein mixtures using isotope-coded affinity tags. *Nat. Biotechnol.* 17 994–999. 10.1038/13690 10504701

[B36] Hall A. Karplus P. A. Poole L. B. (2009). Typical 2-Cys peroxiredoxins – structures, mechanisms and functions. *FEBS J.* 276 2469–2477. 10.1111/j.1742-4658.2009.06985.x 19476488 PMC2747500

[B37] Held J. M. Danielson S. R. Behring J. B. Atsriku C. Britton D. J. Puckett R. L. (2010). Targeted quantitation of site-specific cysteine oxidation in endogenous proteins using a differential alkylation and multiple reaction monitoring mass spectrometry approach. *Mol. Cell. Proteomics* 9 1400–1410. 10.1074/mcp.m900643-mcp200 20233844 PMC2938085

[B38] Ising C. Venegas C. Zhang S. Scheiblich H. Schmidt S. V. Vieira-Saecker A. (2019). NLRP3 inflammasome activation drives tau pathology. *Nature* 575 669–673. 31748742 10.1038/s41586-019-1769-z PMC7324015

[B39] Jaffrey S. R. Erdjument-Bromage H. Ferris C. D. Tempst P. Snyder S. H. (2001). Protein S-nitrosylation: a physiological signal for neuronal nitric oxide. *Nat. Cell. Biol.* 3 193–197. 10.1038/35055104 11175752

[B40] Jones D. P. (2006). Redefining oxidative stress. *Antioxid. Redox. Signal.* 8 1865–1879. 10.1089/ars.2006.8.1865 16987039

[B41] Joshi A. U. Minhas P. S. Liddelow S. A. Haileselassie B. Andreasson K. I. Dorn G. W. (2019). Fragmented mitochondria released from microglia trigger A1 astrocytic response and propagate inflammatory neurodegeneration. *Nat. Neurosci.* 22 1635–1648. 10.1038/s41593-019-0486-0 31551592 PMC6764589

[B42] Kolb H. C. Finn M. G. Sharpless K. B. (2001). Click chemistry: diverse chemical function from a few good reactions. *Angew. Chem. Int. Ed. Engl.* 40 2004–2021. 10.1002/1521-3773(20010601)40:11<2004::aid-anie2004>3.0.co;2-5 11433435

[B43] Konat G. W. Wiggins R. C. (1985). Effect of reactive oxygen species on myelin membrane proteins. *J. Neurochem.* 45 1113–1118. 10.1111/j.1471-4159.1985.tb05530.x 4031880

[B44] Lange V. Picotti P. Domon B. Aebersold R. (2008). Selected reaction monitoring for quantitative proteomics: a tutorial. *Mol. Syst. Biol.* 4:222. 10.1038/msb.2008.61 18854821 PMC2583086

[B45] Lee C.-F. Paull T. T. Person M. D. (2013). Proteome-wide detection and quantitative analysis of irreversible cysteine oxidation using long column UPLC-pSRM. *J. Proteome Res.* 12 4302–4315. 10.1021/pr400201d 23964713 PMC3823053

[B46] Li F. Calingasan N. Y. Yu F. Mauck W. M. Toidze M. Almeida C. G. (2004). Increased plaque burden in brains of APP mutant MnSOD heterozygous knockout mice. *J. Neurochem.* 89 1308–1312. 10.1111/j.1471-4159.2004.02455.x 15147524

[B47] Liedhegner E. A. S. Gao X.-H. Mieyal J. J. (2012). Mechanisms of altered redox regulation in neurodegenerative diseases—Focus on S-glutathionylation. *Antioxidants Redox Signal.* 16 543–566. 10.1089/ars.2011.4119 22066468 PMC3270051

[B48] Lin M. T. Beal M. F. (2006). Mitochondrial dysfunction and oxidative stress in neurodegenerative diseases. *Nature* 443 787–795. 10.1038/nature05292 17051205

[B49] Lorenzatto K. R. Kim K. Ntai I. Paludo G. P. Camargo De Lima J. Thomas P. M. (2015). Top down proteomics reveals mature proteoforms expressed in subcellular fractions of the *Echinococcus granulosus* preadult stage. *J. Proteome Res.* 14 4805–4814. 10.1021/acs.jproteome.5b00642 26465659 PMC4638118

[B50] Markesbery W. R. Carney J. M. (1999). Oxidative alterations in Alzheimer’s disease. *Brain Pathol.* 9 133–146. 9989456 10.1111/j.1750-3639.1999.tb00215.x PMC8098393

[B51] Maurais A. J. Weerapana E. (2019). Reactive-cysteine profiling for drug discovery. *Curr. Opin. Chem. Biol.* 50 29–36. 10.1016/j.cbpa.2019.02.010 30897495 PMC6584045

[B52] McConnell E. W. Smythers A. L. Hicks L. M. (2020). Maleimide-based chemical proteomics for quantitative analysis of cysteine reactivity. *J. Am. Soc. Mass Spectrometry* 31 1697–1705. 10.1021/jasms.0c00116 32573231

[B53] McDonagh B. Sakellariou G. K. Smith N. T. Brownridge P. Jackson M. J. (2014). Differential cysteine labeling and global label-free proteomics reveals an altered metabolic state in skeletal muscle aging. *J. Proteome Res.* 13 5008–5021. 10.1021/pr5006394 25181601 PMC4227305

[B54] Meyer J. G. Schilling B. (2017). Clinical applications of quantitative proteomics using targeted and untargeted data-independent acquisition techniques. *Expert Rev. Proteomics* 14 419–429. 10.1080/14789450.2017.1322904 28436239 PMC5671767

[B55] Mnatsakanyan R. Markoutsa S. Walbrunn K. Roos A. Verhelst S. H. L. Zahedi R. P. (2019). Proteome-wide detection of S-nitrosylation targets and motifs using bioorthogonal cleavable-linker-based enrichment and switch technique. *Nat. Commun.* 10:2195. 10.1038/s41467-019-10182-4 PMC6522481 31097712

[B56] Murray C. I. Eyk J. E. V. (2012). Chasing cysteine oxidative modifications: proteomic tools for characterizing cysteine redox status. *Circ. Cardiovasc. Genet.* 5:591. 10.1161/circgenetics.111.961425 23074338 PMC3500588

[B57] Nakamura A. Kaneko N. Villemagne V. L. Kato T. Doecke J. Doré V. (2018). High performance plasma amyloid-β biomarkers for Alzheimer’s disease. *Nature* 554 249–254. 29420472 10.1038/nature25456

[B58] Nakamura T. Lipton S. A. (2007). S-Nitrosylation and uncompetitive/fast off-rate (UFO) drug therapy in neurodegenerative disorders of protein misfolding. *Cell. Death Differ.* 14 1305–1314. 10.1038/sj.cdd.4402138 17431424

[B59] Nakamura T. Tu S. Akhtar M. W. Sunico C. R. Okamoto S. Lipton S. A. (2013). Aberrant protein s-nitrosylation in neurodegenerative diseases. *Neuron* 78 596–614. 10.1016/j.neuron.2013.05.005 23719160 PMC3712898

[B60] Newman S. F. Sultana R. Perluigi M. Coccia R. Cai J. Pierce W. M. (2007). An increase in S-glutathionylated proteins in the Alzheimer’s disease inferior parietal lobule, a proteomics approach. *J. Neurosci. Res.* 85 1506–1514. 10.1002/jnr.21275 17387692

[B61] Olzscha H. (2019). Posttranslational modifications and proteinopathies: how guardians of the proteome are defeated. *Biol. Chem.* 400 895–915. 10.1515/hsz-2018-0458 30998500

[B62] Pappireddi N. Martin L. Wühr M. (2019). A review on quantitative multiplexed proteomics. *Chembiochem* 20 1210–1224. 10.1002/cbic.201800650 30609196 PMC6520187

[B63] Papuć E. Kurys-Denis E. Krupski W. Tatara M. Rejdak K. (2015). Can antibodies against glial derived antigens be early biomarkers of hippocampal demyelination and memory loss in Alzheimer’s disease? *J. Alzheimer’s Dis.* 48 115–121. 10.3233/jad-150309 26401933

[B64] Parker J. Balmant K. Zhu F. Zhu N. Chen S. (2015). cysTMTRAQ-An integrative method for unbiased thiol-based redox proteomics. *Mol. Cell. Proteomics* 14 237–242. 10.1074/mcp.o114.041772 25316711 PMC4288258

[B65] Paula A. Rodrigo A. C. Catarina O. (2010). Neuroinflammation, oxidative stress and the pathogenesis of Alzheimers disease. *Curr. Pharm. Design* 16 2766–2778. 10.2174/138161210793176572 20698820

[B66] Paulech J. Solis N. Edwards A. V. Puckeridge M. White M. Y. Cordwell S. J. (2013). Large-scale capture of peptides containing reversibly oxidized cysteines by thiol-disulfide exchange applied to the myocardial redox proteome. *Anal. Chem.* 85 3774–3780. 10.1021/ac400166e 23438843

[B67] Percy A. J. Chambers A. G. Yang J. Hardie D. B. Borchers C. H. (2014). Advances in multiplexed MRM-based protein biomarker quantitation toward clinical utility. *Biochim. Biophys. Acta* 1844 917–926. 10.1016/j.bbapap.2013.06.008 23806606

[B68] Poulsen K. Bahl J. M. Simonsen A. H. Hasselbalch S. G. Heegaard N. H. (2014). Distinct transthyretin oxidation isoform profile in spinal fluid from patients with Alzheimer’s disease and mild cognitive impairment. *Clin. Proteomics* 11:12. 10.1186/1559-0275-11-12 24678637 PMC3973606

[B69] Puspita L. Chung S. Y. Shim J. W. (2017). Oxidative stress and cellular pathologies in Parkinson’s disease. *Mol. Brain* 10:53. 10.1186/s13041-017-0340-9 PMC5706368 29183391

[B70] Qian Y. Weerapana E. (2017). “A quantitative mass-spectrometry platform to monitor changes in cysteine reactivity,” in *Activity-based proteomics: methods and protocols*, eds Overkleeft H. S. Florea B. I. (New York City, NY: Springer), 11–22. 10.1007/978-1-4939-6439-0_2 PMC5359601 27778278

[B71] Qu Z. Meng F. Bomgarden R. D. Viner R. I. Li J. Rogers J. C. (2014). Proteomic quantification and site-mapping of S-nitrosylated proteins using isobaric iodoTMT reagents. *J. Proteome Res.* 13 3200–3211. 10.1021/pr401179v 24926564 PMC4084841

[B72] Redler R. L. Wilcox K. C. Proctor E. A. Fee L. Caplow M. Dokholyan N. V. (2011). Glutathionylation at Cys-111 induces dissociation of wild type and FALS mutant SOD1 dimers. *Biochemistry* 50 7057–7066. 10.1021/bi200614y 21739997 PMC3281512

[B73] Reinwarth M. Avrutina O. Fabritz S. Kolmar H. (2014). Fragmentation follows structure: top-down mass spectrometry elucidates the topology of engineered cystine-knot miniproteins. *PLoS One* 9:e108626. 10.1371/journal.pone.0108626 25303319 PMC4193770

[B74] Seneviratne U. Nott A. Bhat V. B. Ravindra K. C. Wishnok J. S. Tsai L.-H. (2016). S-nitrosation of proteins relevant to Alzheimer’s disease during early stages of neurodegeneration. *Proc. Natl. Acad. Sci.* 113:4152. 10.1073/pnas.1521318113 27035958 PMC4839463

[B75] Shannon D. A. Banerjee R. Webster E. R. Bak D. W. Wang C. Weerapana E. (2014). Investigating the proteome reactivity and selectivity of aryl halides. *J. Am. Chem. Soc.* 136 3330–3333. 10.1021/ja4116204 24548313

[B76] Shao B.-Z. Cao Q. Liu C. (2018). Targeting NLRP3 inflammasome in the treatment of CNS Diseases. *Front. Mol. Neurosci.* 11:320. 10.3389/fnmol.2018.00320 30233319 PMC6131647

[B77] Sherrod S. D. Myers M. V. Li M. Myers J. S. Carpenter K. L. Maclean B. (2012). Label-free quantitation of protein modifications by pseudo selected reaction monitoring with internal reference peptides. *J. Proteome Res.* 11 3467–3479. 10.1021/pr201240a 22559222 PMC3368409

[B78] Shi Y. Carroll K. S. (2020). Activity-based sensing for site-specific proteomic analysis of cysteine oxidation. *Acc. Chem. Res.* 53 20–31. 10.1021/acs.accounts.9b00562 31869209 PMC7061859

[B79] Song M. Zhao X. Song F. (2021). Aging-dependent mitophagy dysfunction in Alzheimer’s disease. *Mol. Neurobiol.* 58 2362–2378. 10.1007/s12035-020-02248-y 33417222

[B80] Srivastava D. Kukkuta Sarma G. R. Dsouza D. S. Muralidharan M. Srinivasan K. Mandal A. K. (2019). Characterization of residue-specific glutathionylation of CSF proteins in multiple sclerosis – A MS-based approach. *Anal. Biochem.* 564-565 108–115. 10.1016/j.ab.2018.10.015 30367882

[B81] Swerdlow R. H. Burns J. M. Khan S. M. (2014). The Alzheimer’s disease mitochondrial cascade hypothesis: progress and perspectives. *Biochim. Biophys. Acta* 1842 1219–1231. 10.1016/j.bbadis.2013.09.010 24071439 PMC3962811

[B82] Swerdlow R. H. Khan S. M. (2004). A “mitochondrial cascade hypothesis” for sporadic Alzheimer’s disease. *Medical Hypotheses* 63 8–20. 10.1016/j.mehy.2003.12.045 15193340

[B83] Tahir M. Nawrocki A. Ditzel H. J. Larsen M. R. (2020). Increasing proteome coverage using cysteine-specific DIA mass spectrometry – Cys-DIA. *bioRxiv* 2020:966861.

[B84] Tian C. Sun R. Liu K. Fu L. Liu X. Zhou W. (2017). Multiplexed thiol reactivity profiling for target discovery of electrophilic natural products. *Cell. Chem. Biol.* 24 1416–1427. 10.1016/j.chembiol.2017.08.022 28988947

[B85] Tran J. C. Zamdborg L. Ahlf D. R. Lee J. E. Catherman A. D. Durbin K. R. (2011). Mapping intact protein isoforms in discovery mode using top-down proteomics. *Nature* 480 254–258. 10.1038/nature10575 22037311 PMC3237778

[B86] van der Reest J. Lilla S. Zheng L. Zanivan S. Gottlieb E. (2018). Proteome-wide analysis of cysteine oxidation reveals metabolic sensitivity to redox stress. *Nat. Commun.* 9:1581. 10.1038/s41467-018-04003-3 PMC5910380 29679077

[B87] Velliquette R. A. O’connor T. Vassar R. (2005). Energy inhibition elevates beta-secretase levels and activity and is potentially amyloidogenic in APP transgenic mice: possible early events in Alzheimer’s disease pathogenesis. *J. Neurosci.* 25 10874–10883. 10.1523/jneurosci.2350-05.2005 16306400 PMC6725876

[B88] Venegas C. Kumar S. Franklin B. S. Dierkes T. Brinkschulte R. Tejera D. (2017). Microglia-derived ASC specks cross-seed amyloid-β in Alzheimer’s disease. *Nature* 552 355–361. 10.1038/nature25158 29293211

[B89] Virág D. Dalmadi-Kiss B. Vékey K. Drahos L. Klebovich I. Antal I. (2020). Current trends in the analysis of post-translational modifications. *Chromatographia* 83 1–10. 10.1007/s10337-019-03796-9

[B90] Wang H. M. Zhang T. Huang J. K. Xiang J. Y. Chen J. Fu J. L. (2017). Edaravone attenuates the proinflammatory response in Amyloid-β-Treated Microglia by inhibiting NLRP3 inflammasome-mediated IL-1β Secretion. *Cell. Physiol. Biochem.* 43 1113–1125. 10.1159/000481753 28977782

[B91] Wang M. You J. Bemis K. G. Tegeler T. J. Brown D. P. (2008). Label-free mass spectrometry-based protein quantification technologies in proteomic analysis. *Brief Funct. Genomic. Proteomic.* 7 329–339. 18579615 10.1093/bfgp/eln031

[B92] Weerapana E. Speers A. E. Cravatt B. F. (2007). Tandem orthogonal proteolysis-activity-based protein profiling (TOP-ABPP)—a general method for mapping sites of probe modification in proteomes. *Nat. Protoc.* 2 1414–1425. 10.1038/nprot.2007.194 17545978

[B93] Weerapana E. Wang C. Simon G. M. Richter F. Khare S. Dillon M. B. D. (2010). Quantitative reactivity profiling predicts functional cysteines in proteomes. *Nature* 468 790–795. 10.1038/nature09472 21085121 PMC3058684

[B94] Wijasa T. S. Sylvester M. Brocke-Ahmadinejad N. Schwartz S. Santarelli F. Gieselmann V. (2020). Quantitative proteomics of synaptosome S-nitrosylation in Alzheimer’s disease. *J. Neurochem.* 152 710–726. 10.1111/jnc.14870 31520481

[B95] Wu Y. Wang F. Liu Z. Qin H. Song C. Huang J. (2014). Five-plex isotope dimethyl labeling for quantitative proteomics. *Chem. Commun.* 50 1708–1710. 10.1039/c3cc47998f 24394284

[B96] Yang F. Gao J. Che J. Jia G. Wang C. (2018). A dimethyl-labeling-lased strategy for site-specifically quantitative chemical proteomics. *Anal. Chem.* 90 9576–9582. 10.1021/acs.analchem.8b02426 29989794

[B97] Zahid S. Khan R. Oellerich M. Ahmed N. Asif A. R. (2014). Differential S-Nitrosylation of proteins in Alzheimer’s disease. *Neuroscience* 256 126–136. 10.1016/j.neuroscience.2013.10.026 24157928

[B98] Zhao Q.-F. Yu J.-T. Tan L. (2015). S-Nitrosylation in Alzheimer’s disease. *Mol. Neurobiol.* 51 268–280. 24664522 10.1007/s12035-014-8672-2

