# Supplementary material for: Proteomic Approaches to Study Cysteine Oxidation: Applications in Neurodegenerative Diseases
Source: Front Mol Neurosci. 2021 Jun 9;14:678837. doi: 10.3389/fnmol.2021.678837 (PMC8219902; doi:10.3389/fnmol.2021.678837)
Supplement: Supplementary file 1 [file Data_Sheet_1.PDF]

Table S1. Different types of Oxi-Cys proteomics approaches.

Method	Type of Oxi-Cys PTMs	Labelling Chemicals/Tags	Approach	MS/MS$^{(n)}$ quantification	Samples	Number of Oxi-Cys sites (peptides/proteins)	References
SICyLIA	Global reversible Cys oxidation	Light ($^{12}C2H4INO$) and heavy ($^{13}C2D_2H_2INO$) iodoacetamide<br><br>NEM to block oxidised Cys	Light and heavy stable isotopic (# 4.0 Da)	MS^1	Mouse cells and kidneys	Mouse cells: 9479 unique Cys-containing<br><br>Peptides (3563 proteins)<br><br>Mouse kidney tissues : 4415 Cys-containing<br><br>Peptides (2168 proteins)	(van der Reest et al., 2018)
DiaAlk	*S*-sulfinylation<br><br>S-sulfenylation	DiaAlk-triazohexanoic acid, $C_{15}H_{25}N_5O_7$<br><br>Light and heavy isotoptic UV Cleavable Biotin-Azide $C_{38}H_{51}N_7O_9S$<br><br>BTD-triazohexanoic acid, $C_{19}H_{22}N_4O_5S$<br><br>Azide–alkyne cycloaddition	Light and heavy isotopic tags (# 6.0201 Da)	MS^1	Hela and A549 cell line	387 probe-tagged S-sulfinylated sites/ 296 proteins<br><br>1,173 and 1,098 S-sulfenylation sites in A549 and HeLa cells	(Akter et al., 2018)

IodoTMT	Global and selective Oxi-Cys	IodoTMT reagents	126, 127, 128, 129, 130 and 131 *m/z*	MS^2	Microglial cells		(Qu et al., 2014)
CysTMTRAQ	Global and selective Oxi-Cys and global proteins' abundance	CysTMT tags and iTRAQ tags	Different in isobaric tags<br>CysTMT: 126, 127, 128, 129, 130 and 131 *m/z*<br>TMT: 114, 115, 116, 117, 118, 119 and 121 *m/z*	MS^2	*Escherichia coli*	912 cysteine-containing peptides	(Parker et al., 2015)
isoTOP-ABPP	proteome-wide surveys of cysteine reactivity	Light and heavy alkynylated IA-probes<br>Idoactamide conating alkene group<br>TEV-biotin tags	Light and heavy isotopic tags (# 6.0201 Da)	MS^1	Yeast, mouse, and human cancer cell lines	1097 Cys-containing peptides detected in human cell lines<br>168 Cys-peptides in mouse heart	(Weerapana et al., 2007;van der Reest et al., 2018)
QTRP	Measuring reversible Cys	IPM, light and heavy (13C6) Az-UV-biotin reagents			Human cell lines	6566 cysteines on 3557 proteins	(van der Reest et al., 2018)
OxcyscPILOT	S-nitrosylation and S-glutathionylation	light for total Cys, or heavy SON PTM) demethylation<br>CH2O and $^{13}CD_2O$	TMT	MS2/MS3	Mouse with AD vs Wt		(Dyer et al., 2017)

		Coupled with TMT or iTRAQ					
OxiMRM	All forms of Cys oxidation	Ligh and heavy NEM (stable isotope-labeled<br>NEM)	LFQ (MRM)<br># mass 5.0314 Da	MS^2, MRM	Human breast cancer	Target proteins (protein p53 and protein tyrosine phosphatase-1B (PTP1B)	(Held et al., 2010)
UPLC-pSRM	All form of Cys oxidation, especially for irreversibly oxidised Cys			MS^1, pSRM	Jurkat cell lines	61 cysteine (sulfonic and sulfinic acid) oxidised peptides identified from 43 proteins	(Sherrod et al., 2012)
Cys-DIA	Global of all Cys	Enrich Cys-containing peptides by Thiopropyl Sepharose™ 6B beads	LFQ	MS1	HeLa and cancer cells	47,657 unique Cys-peptides, covering 8,698 proteins	(Tahir et al., 2020)

References

Van Der Reest, J., Lilla, S., Zheng, L., Zanivan, S., and Gottlieb, E. (2018). Proteome-wide analysis of cysteine oxidation reveals metabolic sensitivity to redox stress. *Nat Commun* 9**,** 1581.

Akter, S., Fu, L., Jung, Y., Conte, M.L., Lawson, J.R., Lowther, W.T., Sun, R., Liu, K., Yang, J., and Carroll, K.S. (2018). Chemical proteomics reveals new targets of cysteine sulfinic acid reductase. *Nature Chemical Biology* 14**,** 995-1004.

Qu, Z., Meng, F., Bomgarden, R.D., Viner, R.I., Li, J., Rogers, J.C., Cheng, J., Greenlief, C.M., Cui, J., Lubahn, D.B., Sun, G.Y., and Gu, Z. (2014). Proteomic quantification and site-mapping of S-nitrosylated proteins using isobaric iodoTMT reagents. *Journal of proteome research* 13**,** 3200-3211.

Parker, J., Balmant, K., Zhu, F., Zhu, N., and Chen, S. (2015). cysTMTRAQ-An integrative method for unbiased thiol-based redox proteomics. *Mol Cell Proteomics* 14**,** 237-242.

Weerapana, E., Speers, A.E., and Cravatt, B.F. (2007). Tandem orthogonal proteolysis-activity-based protein profiling (TOP-ABPP)—a general method for mapping sites of probe modification in proteomes. *Nature Protocols* 2**,** 1414-1425.

Van Der Reest, J., Lilla, S., Zheng, L., Zanivan, S., and Gottlieb, E. (2018). Proteome-wide analysis of cysteine oxidation reveals metabolic sensitivity to redox stress. *Nat Commun* 9**,** 1581.

Dyer, R.R., Gu, L., and Robinson, R.a.S. (2017). "S-nitrosylation in Alzheimer's disease using oxidized cysteine-selective cPILOT," in *Current proteomic approaches applied to brain function,* eds. E. Santamana & J. Fernández-Irigoyen. (New York: Springer New York), 225–241.

Held, J.M., Danielson, S.R., Behring, J.B., Atsriku, C., Britton, D.J., Puckett, R.L., Schilling, B., Campisi, J., Benz, C.C., and Gibson, B.W. (2010). Targeted quantitation of site-specific cysteine oxidation in endogenous proteins using a differential alkylation and multiple reaction monitoring mass spectrometry approach. *Molecular & cellular proteomics : MCP* 9**,** 1400-1410.

Sherrod, S.D., Myers, M.V., Li, M., Myers, J.S., Carpenter, K.L., Maclean, B., Maccoss, M.J., Liebler, D.C., and Ham, A.-J.L. (2012). Label-free quantitation of protein modifications by pseudo selected reaction monitoring with internal reference peptides. *Journal of Proteome Research* 11**,** 3467-3479.

Tahir, M., Nawrocki, A., Ditzel, H.J., and Larsen, M.R. (2020). Increasing proteome coverage using cysteine-specific DIA mass spectrometry – Cys-DIA. *bioRxiv***,** 2020.2002.2027.966861.
