# Supplementary material for: Proteomic Approaches to Study Cysteine Oxidation: Applications in Neurodegenerative Diseases
Source: Front Mol Neurosci. 2021 Jun 9;14:678837. doi: 10.3389/fnmol.2021.678837 (PMC8219902; doi:10.3389/fnmol.2021.678837)
Supplement: Supplementary file 2 [file Data_Sheet_2.PDF]

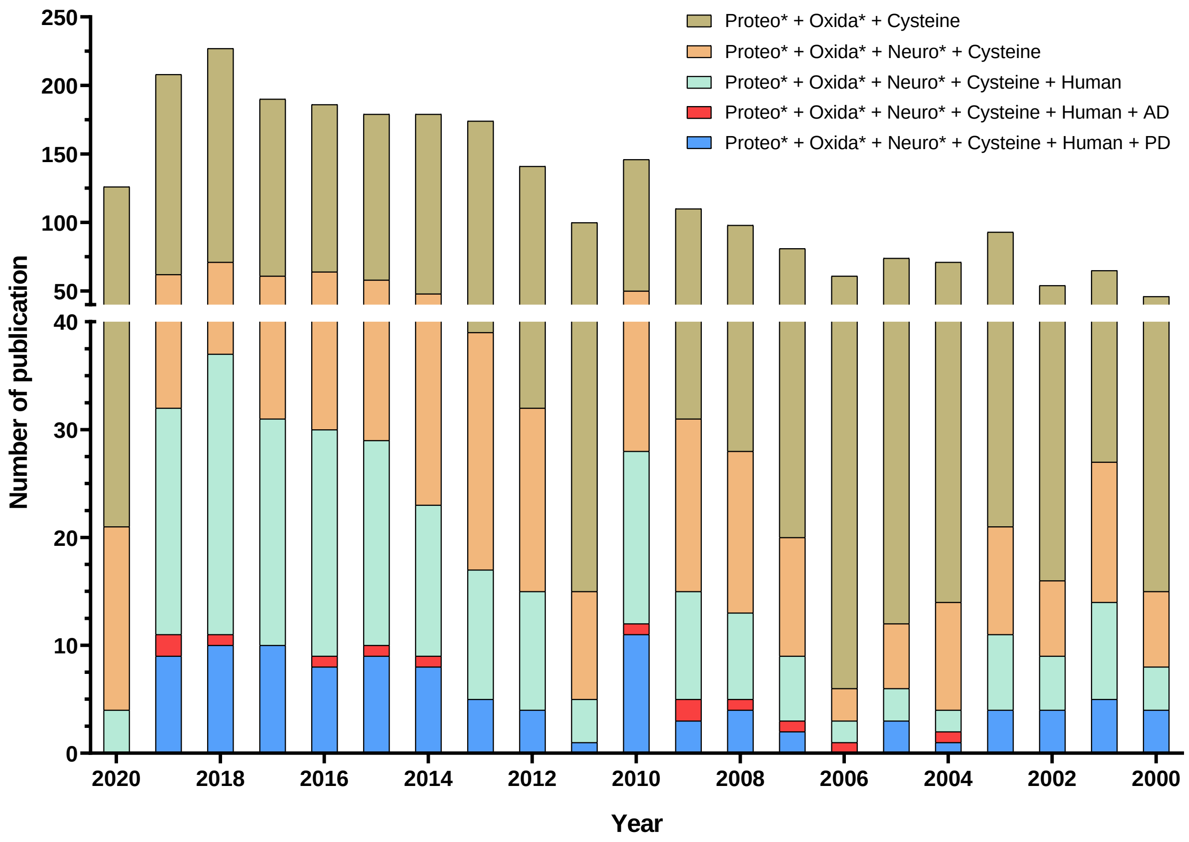

Figure S1. Number of publications relating to Oxi-Cys proteomics available at PubMed. Different combinations of keywords were used: proteo* and oxida* and cysteine (used as fixed keywords), and then other variables such as neuro*, human, AD, PD, MS, and MND (ALS).

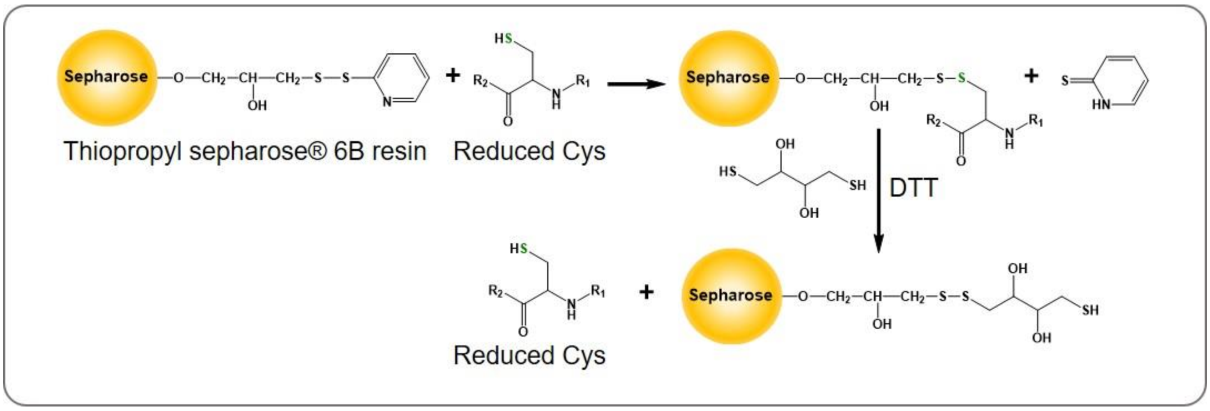

Figure S2. Purification of reduced Cys using Thiopropyl sepharose® 6B resin in the RAC method.
